# Motion correction for routine X-ray lung CT imaging

**DOI:** 10.1038/s41598-021-83403-w

**Published:** 2021-02-12

**Authors:** Doil Kim, Jiyoung Choi, Duhgoon Lee, Hyesun Kim, Jiyoung Jung, Minkook Cho, Kyoung-Yong Lee

**Affiliations:** CT R&D Group, Health & Medical Equipment Business, Samsung Electronics Co., Ltd., Suwon, Republic of Korea

**Keywords:** Biomedical engineering, Preclinical research

## Abstract

A novel motion correction algorithm for X-ray lung CT imaging has been developed recently. It was designed to perform for routine chest or thorax CT scans without gating, namely axial or helical scans with pitch around 1.0. The algorithm makes use of two conjugate partial angle reconstruction images for motion estimation via non-rigid registration which is followed by a motion compensated reconstruction. Differently from other conventional approaches, no segmentation is adopted in motion estimation. This makes motion estimation of various fine lung structures possible. The aim of this study is to explore the performance of the proposed method in correcting the lung motion artifacts which arise even under routine CT scans with breath-hold. The artifacts are known to mimic various lung diseases, so it is of great interest to address the problem. For that purpose, a moving phantom experiment and clinical study (seven cases) were conducted. We selected the entropy and positivity as figure of merits to compare the reconstructed images before and after the motion correction. Results of both phantom and clinical studies showed a statistically significant improvement by the proposed method, namely up to 53.6% (p < 0.05) and up to 35.5% (p < 0.05) improvement by means of the positivity measure, respectively. Images of the proposed method show significantly reduced motion artifacts of various lung structures such as lung parenchyma, pulmonary vessels, and airways which are prominent in FBP images. Results of two exemplary cases also showed great potential of the proposed method in correcting motion artifacts of the aorta which is known to mimic aortic dissection. Compared to other approaches, the proposed method provides an excellent performance and a fully automatic workflow. In addition, it has a great potential to handle motions in wide range of organs such as lung structures and the aorta. We expect that this would pave a way toward innovations in chest and thorax CT imaging.

## Introduction

Motion artifacts of lung structures are still unavoidable in routine practice^1–5^ of chest CT scans. It was pointed out that pulsatile motion of the heart or involuntary motion of the diaphragm are the main cause of the lung motion happening even under breath-hold during CT scans^1,2^. When it comes to the cardiac-induced lung motion, it was reported that it reaches 1 to 4 mm^4^ or 0.2 to 2.6 mm^5^. As a result, motion artifacts of various lung structures such as lung parenchyma, pulmonary vessels or airways are often visible in routine CT images. These artifacts impose challenges in the diagnosis of lung using CT, since they mimic various lung diseases, including bronchiectasis due to doubling edges, cyst, emphysema, or ground glass opacity (GGO) nodule because of CT-value bias^1,2^. In addition, motion artifacts are one of the challenges in quantitative chest CT^6^.

A breathing motion correction algorithm was proposed before which was based on slab-wise rigid motion estimation^7^. However, this would not be ideal in handling lung motion which is different region by region even in the same slice location^1–3^. We proposed a novel lung motion correction algorithm^8,9^, sub-cycle universal linear model low-dose imaging for thorax (SCULLI-TX), for non-gating chest CT scans and demonstrated the feasibility using XCAT simulation. More specifically, an adaptive parameter setting in motion estimation depending on the motion of the target organ was proposed in^8^ and a preliminary result via XCAT simulation of free breathing CT scans was introduced in^9^. No quantitative evaluation of the SCULLI-TX based on real raw data acquired from CT systems has been reported so far. In this paper, we introduce the results of studies based on a physically moving phantom and human subjects in which the novelty of this paper lies. Unlike the idea^7^ adopting a pure image-based metric and considering only rigid motion, we make use of two reference images and estimate non-rigid motion. These images could be complete or incomplete depending on the target organ or scan conditions^8,9^. We are of the opinion that the motion estimation based on two reference images could be a better option over a pure image-based approach, since two complete or incomplete images could provide motion information and guide the search for the direction and amount of motion. The concept of making use of two conjugate partial angle reconstruction (PAR) images and its efficacy in cardiac motion correction were already reported in literature^10–15^. The proposed method serves as an extension of the original concept^10,11^ to non-gating CT scans and deals with lung and thoracic motions. Handling other cardiac motion correction algorithms would be beyond the scope of this paper. Some related works can be found in our latest study^15^.

Now, we shortly introduce the key concept of SCULLI-TX. For a given raw data we apply fan to parallel rebinning first. For each slice location (or z-position), we have a time point defined which corresponds to the acquisition time of the middle of the raw data which is used for the reconstruction. This time point at each slice location serves as a target time point in motion estimation and correction. Using the first and last segments of the raw data we get two PAR images at the same slice location which are conjugate, namely 90 degrees before and after the target time point. The length and the time difference of the segments can be adjusted in a flexible manner according to the application^8,9^. In this paper, we fixed the length of the segment for PAR images heuristically to the fan angle of a prototype CT scanner to deal with the heart-induced motion. In the case of helical scan, we generate pairs of PAR image at each slice location. We assume that the helical pitch is not larger than 1.0 so that PAR pairs capture the same structural information. These two PAR image stacks form two PAR image volumes in the end. We apply a band-pass filtering to the PAR image volumes in order to discard non-structural information such as shadings. Motion estimation is then carried out by help of non-rigid registration using two PAR image volumes. Here, we adopted a free form deformation which is based on B-splines^16^. Since we have no segmentation step, motion of all edges in PAR images is measured. This way we can deal with motion of various lung structures such as lung parenchyma, pulmonary vessels and airways. Now we have a motion vector field (MVF) describing the motion between two PAR image volumes. By assuming a linear behavior, we can acquire MVF at each view. Lastly, motion compensated reconstruction is performed resulting in time-resolved image volume. We adopted an FBP-type reconstruction to provide a natural noise appearance that is favorable for radiologists. For more details, please refer to the previous works^8,9,15^.

The aim of our study is to explore the capability of SCULLI-TX in correcting motion artifacts of lung and thoracic structures which arise even under routine CT scans with breath-hold. Both phantom and clinical studies were conducted. We adopted entropy and normalized positivity as a measure to quantify motion artifacts. Paired t-test was conducted and p < 0.05 was considered to be statistically significant. To explore the capability of motion correction in other thoracic structures such as the aorta and the superior vena cava (SVC), we investigated the images of FBP and SCULLI-TX qualitatively. A prototype 128 slice CT scanner was utilized for both studies.

## Methods

**Figure 1:**
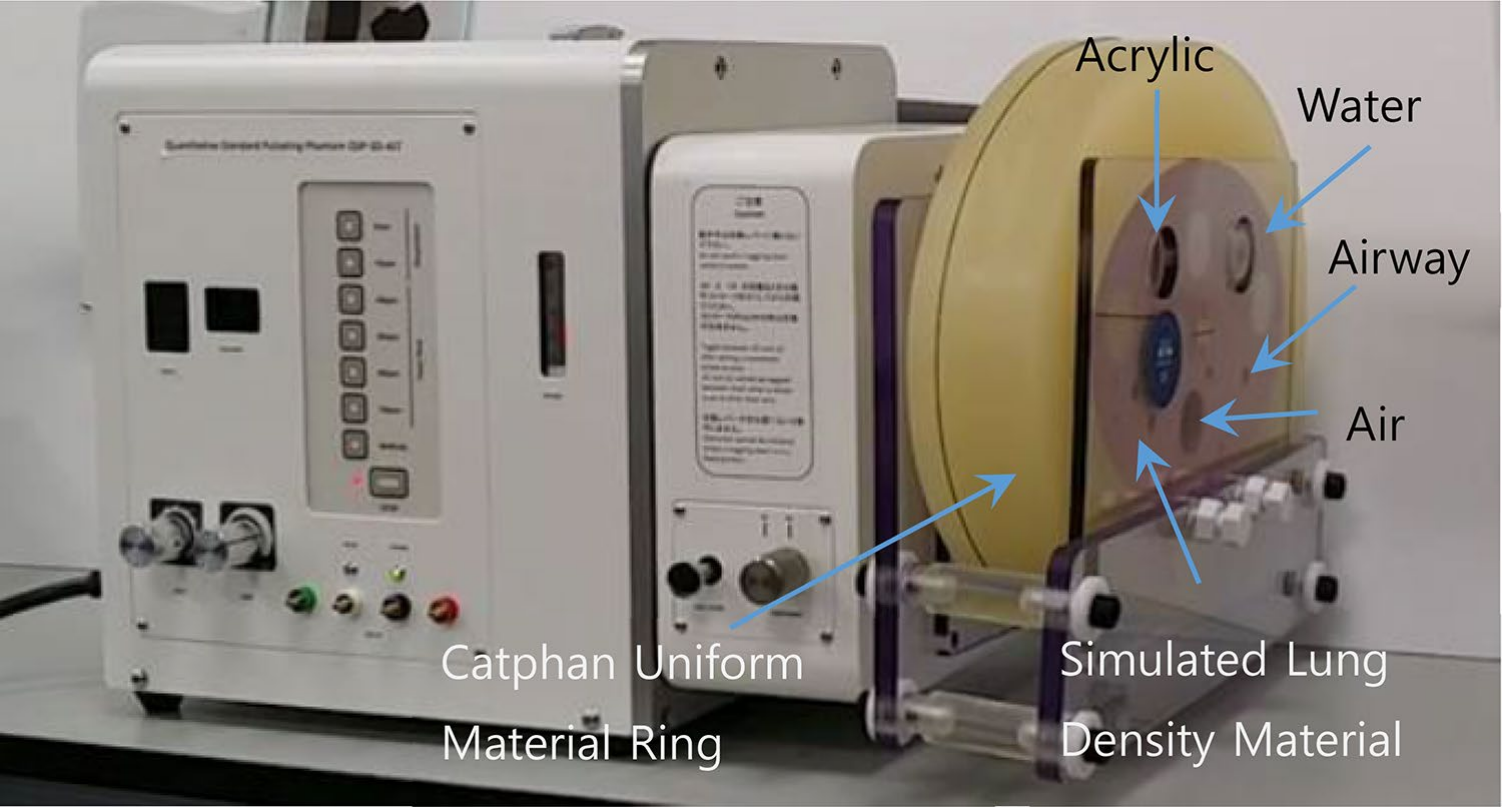
Experimental set-up of the COPD gene phantom. It was attached to the QSP actuator.

We conducted a phantom experiment and a clinical study to explore the performance of SCULLI-TX. For the phantom study we used COPD gene phantom (CTP699, Phantom Laboratory, USA) after attaching it to a motion actuator (QSP-3D-ALT, Fuyo, Japan) as in Fig. 1. We imposed in-plane translational motion and scanned it in an axial scan mode on a prototype CT scanner. The clinical study was IRB approved (number: SMC 2015-06-066-001) in Samsung Medical Center (SMC). The study was performed in accordance with GCP (good clinical research practice). A written informed consent was acquired from all participants. The scan protocol was standard helical scan with breath-hold.

We selected two measures as figure of merits, namely entropy and positivity which have been utilized in other studies^7,17^. First, the entropy has been utilized to quantify motion artifacts in CT images and defined as below:

$$\mathrm{Entropy}= -\sum_{h}P\left(h\right)\mathrm{ln}P(h)$$

where $\mathrm{h}$ is the CT-value, $P(h)$ is the probability density function of a CT-value of $\mathrm{h}$. For the calculation of $P(h)$ we used the kernel density estimation using Parzen windowing with a Gaussian kernel. In our study, we specified a region of interest (ROI) in CT images and calculated $P\left(h\right)$ using the pixel values in the ROI. On the other hand, positivity is another measure to quantify the power of pixel intensities under a certain threshold. Positivity in a specific ROI $\Omega$ with the threshold $\mathrm{T}$ is defined below.

$$\mathrm{Positivity}= {\sum }_{X\in \Omega }\left(\left\{\begin{array}{ll}0,& f\left(X\right)>T\\ {\left(f\left(X\right)-T\right)}^{2},& otherwise\end{array}\right.\right)$$

Here, we automatically calculated $\mathrm{T}$ for a given ROI by choosing a CT-value which maximize the probability $P\left(h\right).$ Note that $\mathrm{f}\left(X\right)$ is the CT-value of reconstructed image at pixel location $\mathrm{X}$. The positivity is known to capture the shadings arising as a result of motion artifacts. It is straightforward that the value of positivity depends on the area of ROI. Since we use different areas of ROIs in our study, we normalize it by the number of pixels in the ROI and call it normalized positivity (NP) with the following definition.

$$\mathrm{Normalized\; Positivity }(\mathrm{NP})=\frac{1}{n(\Omega )}{\sum }_{X\in \Omega }\left(\left\{\begin{array}{ll}0,& f\left(X\right)>T\\ {\left(f\left(X\right)-T\right)}^{2},& otherwise\end{array}\right.\right),$$

where $n(\Omega )$ denotes the number of pixels in $\Omega .$

### Phantom experiment

**Figure 2:**
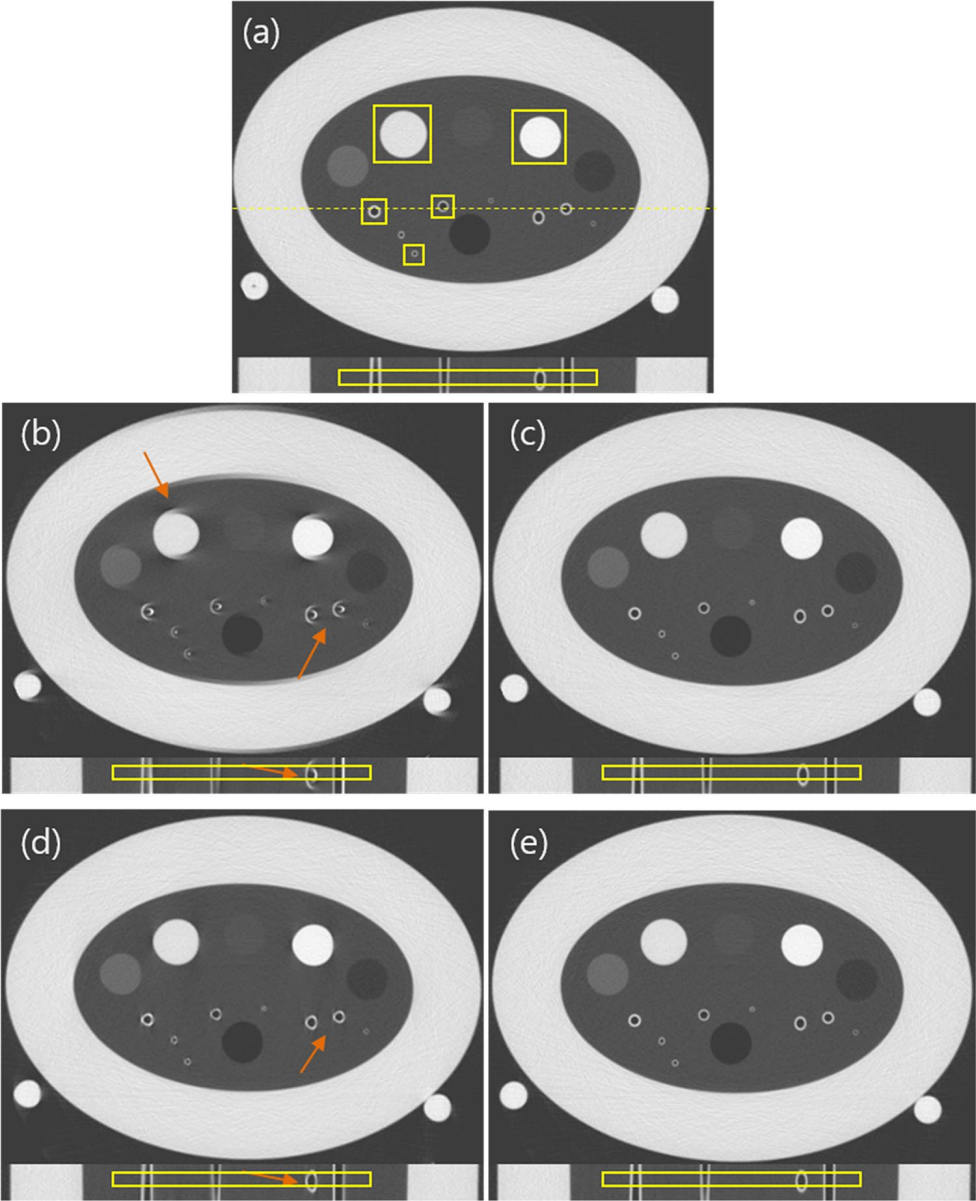
Results of the COPD phantom experiment (magnification 51% for all cases). (a) FBP images in a static status. Transaxial and coronal images along the dotted yellow line. The five ROIs for the calculation of the quantitative measure are marked by the yellow boxes with the solid line. (b) FBP images of 15 RPM case. Orange arrows indicate the motion artifacts appearing as CT-value bias and distorted structures. These have been resolved by SCULLI-TX in (c). (d) FBP images of 10 RPM case. Slight distortion of the structures is present. (e) SCULLI-TX images of 10 RPM case without such distortion. L/W: − 600/1500 HU.

The COPD gene phantom is a lung simulating phantom with air columns and polycarbonate tubes (airway tubes) with various wall thicknesses and angles. It was attached to the motion actuator which can generate periodic motion in one dimension (1D) or three dimensions (3D) by controlling x, y, and z-directional motions (see Fig. 1). The range of vertical stroke is from − 20 to + 20 mm. For axial and longitudinal motion, it is capable of moving from − 7.5 to + 7.5 mm. It offers various cycles controlled by values of rpm (revolution per minute). For our experiment, we applied 1D motion to COPD gene phantom with 0, 5, 10, and 15 rpms under the default setting of the motion. 1D motion is generated by controlling only x-, and y-direction motions. Scans were taken under axial scan mode with 120 kVp, 200 mA, 0.5 s/rot, and 40 mm collimation at a prototype CT scanner. If we combine the vertical and horizontal stroke, we have 42.7 mm for each cycle of the motion. Converting it to the length of the motion per rotation (0.5 s) results in 3.6 mm (5 rpm), 7.1 mm (10 rpm) and 10.7 mm (15 rpm), respectively. The travel lengths of 5 and 10 rpms match with the cardiac-induced lung motion in^4,5^, whereas that of 15 rpm is far beyond it so imposing very challenging condition. Images were reconstructed by FBP (half reconstruction) and SCULLI-TX with 360 mm DFOV, 0.625 mm slice thickness, and standard kernel. Five ROIs marked in Fig. 2 were taken to evaluate motion artifacts by two quantitative measures. We selected a sub-volume of length 10.625 mm (0.625 × 17 slices), which is common in the images of FBP and SCULLI-TX and calculated the measures at each slice on the same ROIs. For each rpm case, we calculated the 17 values from the images of FBP and SCULLI-TX on the area $\Omega =\bigcup_{i=1}^{5}{\Omega }_{i}$ where ${\Omega }_{i}$ denotes the area of $i$ th ROI, $i=1,\dots ,5$. Paired t-test was performed and p < 0.05 was considered to be statistically significant. This way we quantified the relative improvement achieved by SCULLI-TX. Furthermore, we estimated the accuracy of the results of SCULLI-TX (5, 10, and 15 rpm cases) by comparing the results to that of the ground truth (0 rpm FBP).

### Clinical study

**Figure 3:**
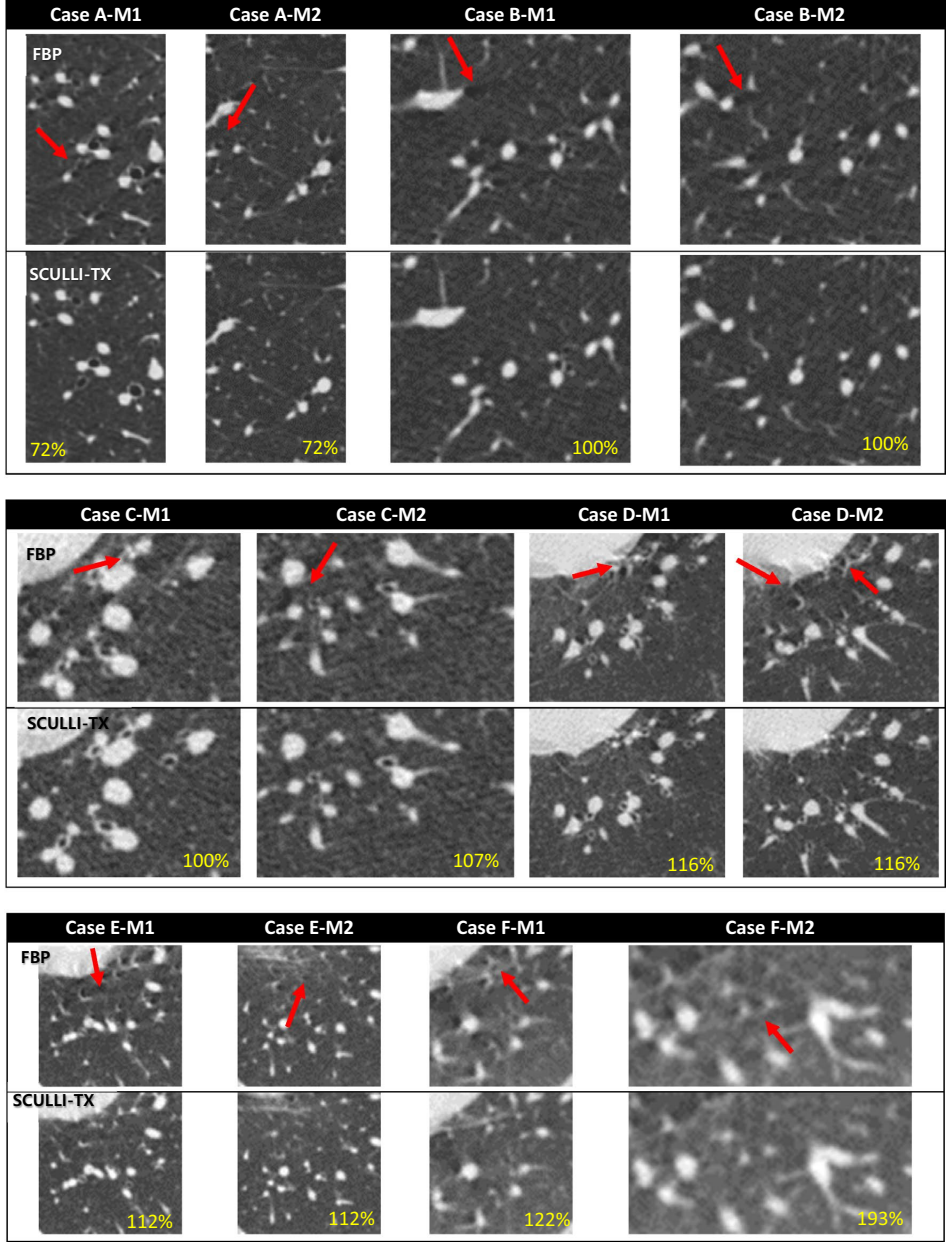
ROIs showing motion artifacts reconstructed by FBP and SCULLI-TX (with the magnification at the bottom of each case provided). Various appearances of lung motion artifacts are observed indicated by the red arrows. For example, seagull or twinkling star-sign (B-M1, C-M1, D-M1, D-M2, E-M1, E-M2, F-M1, F-M2), shadings just beside the vessel or parenchyma which are suffering from motion artifacts (A-M1, A-M2, B-M1, B-M2, C-M1, C-M2, D-M2, E-M1, E-M2, F-M1, F-M2), or distorted airways (A-M1, A-M2, C-M1, C-M2, D-M1, D-M2) are observed in FBP images. These are significantly alleviated in images of SCULLI-TX. L/W: − 500/1500 HU.

**Figure 4:**
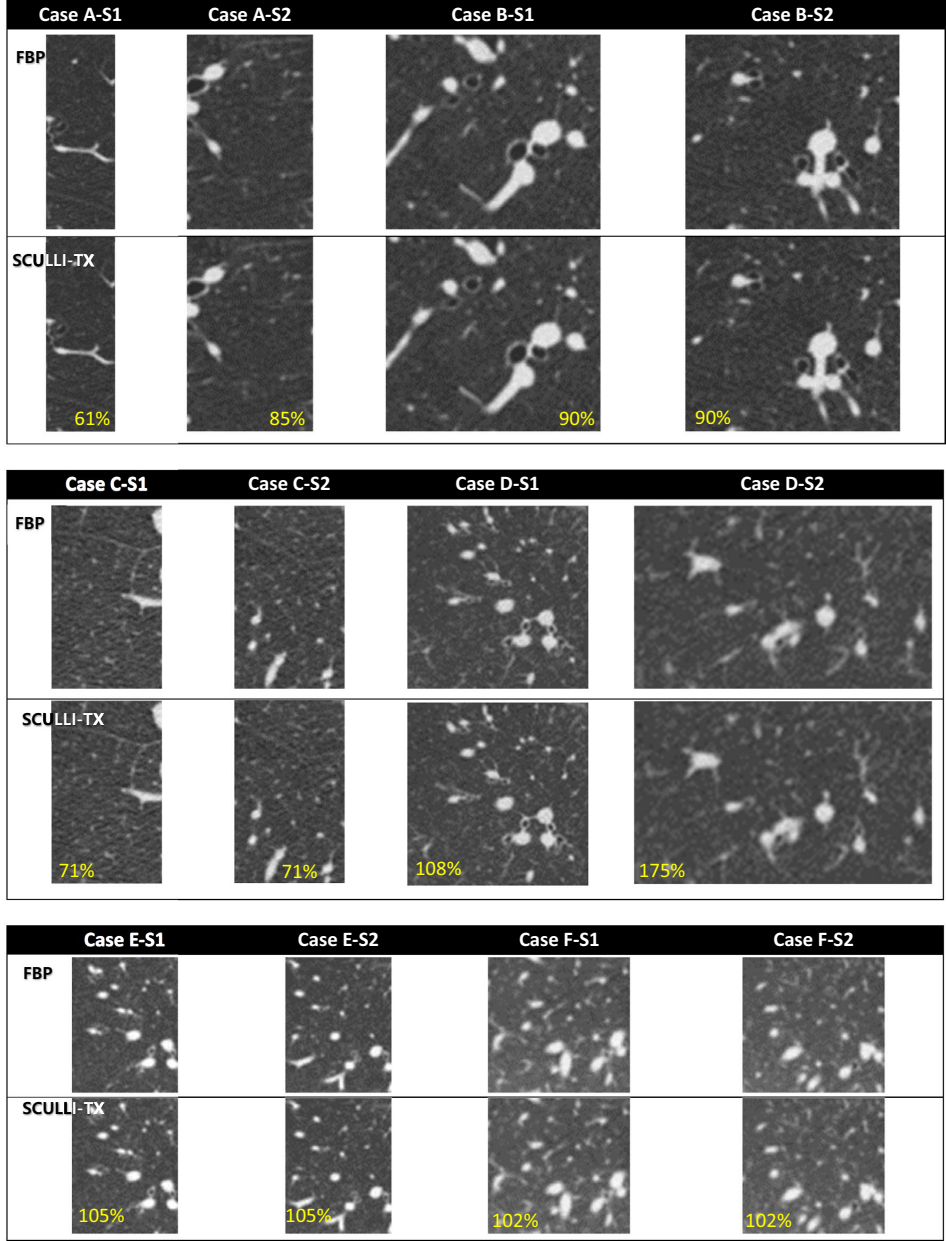
ROIs showing no noticeable motion artifacts reconstructed by FBP and SCULLI-TX (with magnification at the bottom of each case). L/W: − 500/1500 HU.

We chose seven cases retrospectively which showed prominent motion artifacts in lung structures. All subjects underwent CT scans using standard CT protocols with breath-hold. No subjects showed prior symptoms of aortic dissection. Detailed scan protocols are provided in Table 1. Note that case D and E are from a single participant. Images were reconstructed by FBP and SCULLI-TX with 0.625 mm slice thickness. For each case, two slice locations with motion artifacts and those with no motion artifacts were selected respectively by consensus of all authors based on FBP images. At each slice location, an ROI was manually selected for quantitative evaluation of the motion artifacts. Namely, we have 12 ROIs where motion artifacts are prominent in FBP image as shown in Fig. 3. The ROIs with motion artifacts are located on the left (8 cases) and right (4 cases) of the heart, where the transmitted motion of the heart is prominent as indicated in^3^. On the other hand, we have another 12 ROIs where no noticeable motion artifacts are present in FBP images as given in Fig. 4. Also, a paired t-test was performed and p < 0.05 was considered statistically significant. The ROIs are provided in Supplementary Information marked in the full transaxial images.

**Table 1:** Scan conditions of the human subjects.

	Scan mode (protocol)	kVp	mA	Rotation speed (sec/rotation)	Collimation	CTDIv (mGy)	Helical pitch
Case A	Helical (non-contrast chest)	120	300	0.25	80 mm (0.625 × 128)	6.74	0.9
Case B	Helical (non-contrast chest)	120	200	0.25	80 mm (0.625 × 128)	4.49	0.9
Case C	Helical (non-contrast chest)	120	100	0.25	80 mm (0.625 × 128)	2.25	0.9
Case D	Helical (non-contrast chest)	120	250	0.5	40 mm (0.625 × 64)	9.87	1.0
Case E	Helical (contrast chest)	120	250	0.5	40 mm (0.625 × 64)	9.87	1.0
Case F	Helical (contrast abdomen)	120	300	0.5	40 mm (0.625 × 64)	11.87	1.0
Case G	Helical (contrast chest)	120	125	0.5	40 mm (0.625 × 64)	4.89	1.0

In addition, motion artifacts of the thoracic structures were also assessed qualitatively using two exemplary contrast enhanced chest CT exams (case E and case G). In particular, visualization of thoracic structures, especially the aorta, was of great interest. The existence of the so-called pseudo-flap mimicking aortic dissection^18,19^ was investigated by using the image volumes obtained from each of FBP and SCULLI-TX.

## Results

### COPD phantom experiment

**Figure 5:**
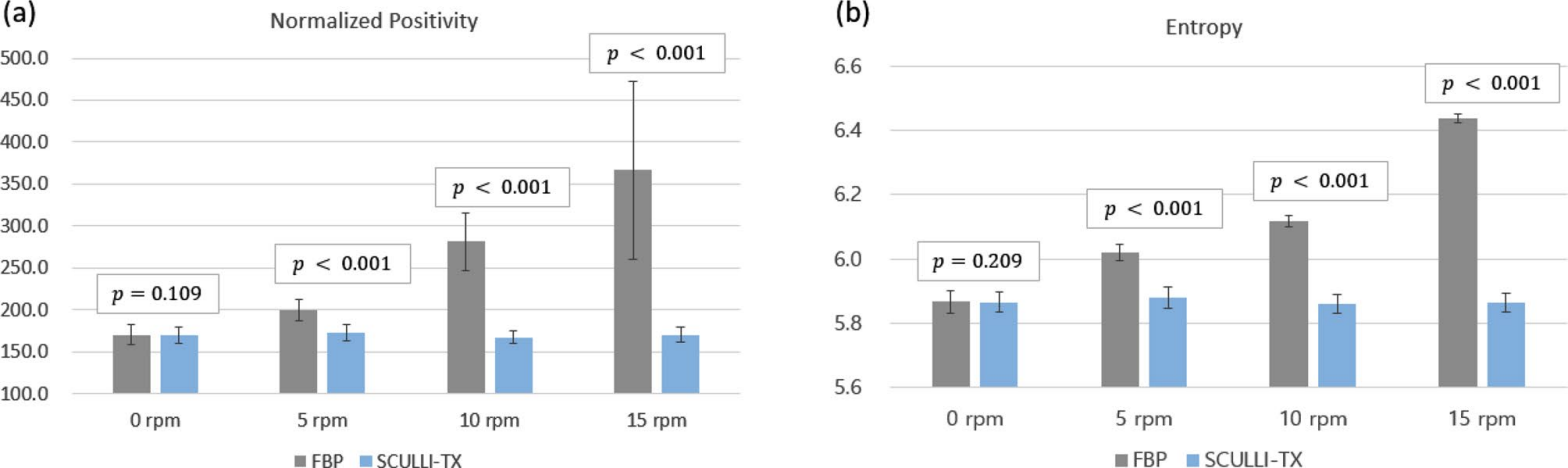
Results of quantitative evaluation of the phantom experiment. It is observed that both measures are, to our expectation, proportional to the severity of the motion. SCULLI-TX shows a statistically significant improvement in terms of both measures as shown in (a) and (b). In 0 rpm case, there was no significant difference between FBP and SCULLI-TX. Comparison of the results of SCULLI-TX to that of the ground truth (0 rpm FBP) is given in Table 2.

Both NP and entropy measures showed a statistically significant improvement by SCULLI-TX as shown in Fig. 5 and Table 2. We start with the results of NP measure. In 0 rpm case, there was no significant difference (FBP: 170.2, SCULLI-TX: 169.2, $\mathrm{p}=0.109$) between NP values of the images of FBP and those of SCULLI-TX as we expected. On the other hand, we achieved 13.6% (5 rpm, FBP: 200.1, SCULLI-TX: 172.9, $\mathrm{p}<0.001$), 40.6% (10 rpm, FBP: 281.6, SCULLI-TX: 167.3, $\mathrm{p}<0.001$), and 53.6% (15 rpm, FBP: 366.4, SCULLI-TX: 169.9, $\mathrm{p}<0.001$) lower NP values by means of SCULLI-TX, which means reduced motion artifacts. The improvement was statistically significant. When it comes to entropy, SCULLI-TX also delivered 2.3% (5 rpm, FBP: 6.019, SCULLI-TX: 5.878, $\mathrm{p}<0.001$), 4.2% (10 rpm, FBP: 6.117, SCULLI-TX: 5.859, $\mathrm{p}<0.001$), and 8.9% (15 rpm, FBP: 6.437, SCULLI-TX: 5.863, $\mathrm{p}<0.001$) lower values, which was also statistically significant. In case of no motion (0 rpm) there was no significant difference (FBP: 5.864, SCULLI-TX: 5.866, $\mathrm{p}=0.209$) too. In Fig. 2, we can see a considerable improvement of SCULLI-TX in terms of alleviated motion artifacts. For example, the CT-value bias near larger structures and the distortion of the airways of FBP in (d) are not observed in the images of SCULLI-TX. In the case of slower motion, namely 10 rpm case, a structural distortion is observed in the FBP image, which was also recovered by SCULLI-TX. This can be appreciated in the result of the quantitative evaluation as depicted in Fig. 5 as well. SCULLI-TX maintains the values of NP and entropy throughout the different motion cases while FBP shows a clear increase of the values being proportional to the severity of the motion. Since the FBP of 0 rpm case is available, we can use it as a ground truth. Comparing results of SCULLI-TX to that of the ground truth reveals no significant difference as provided in Table 2. In other words, SCULLL-TX recovered the motion contaminated images to a level of a static status. The difference images demonstrating the performance of the proposed are provided in Supplementary Fig. S1.

**Table 2:** Comparison to the ground truth (0 rpm FBP) of two quantitative measures, respectively. Both normalized positivity and entropy values of SCULLI-TX show a good match to the ground truth in terms of the small errors and p-values.

	Normalized positivity			Entropy		
	Average (std)	Error	p-value	Average (std)	Error	p-value
0 rpm FBP	170.2 (11.5)	N.A	N.A	5.866 (0.034)	N.A	N.A
5 rpm SCULLI-TX	172.9 (10.2)	+ 1.6%	0.575	5.878 (0.026)	+ 0.2%	0.437
10 rpm SCULLI-TX	167.3 (8.0)	− 1.7%	0.507	5.859 (0.019)	− 0.1%	0.637
15 rpm SCULLI-TX	169.9 (8.9)	− 0.2%	0.944	5.863 (0.014)	0.0%	0.846

### Clinical study

**Figure 6:**
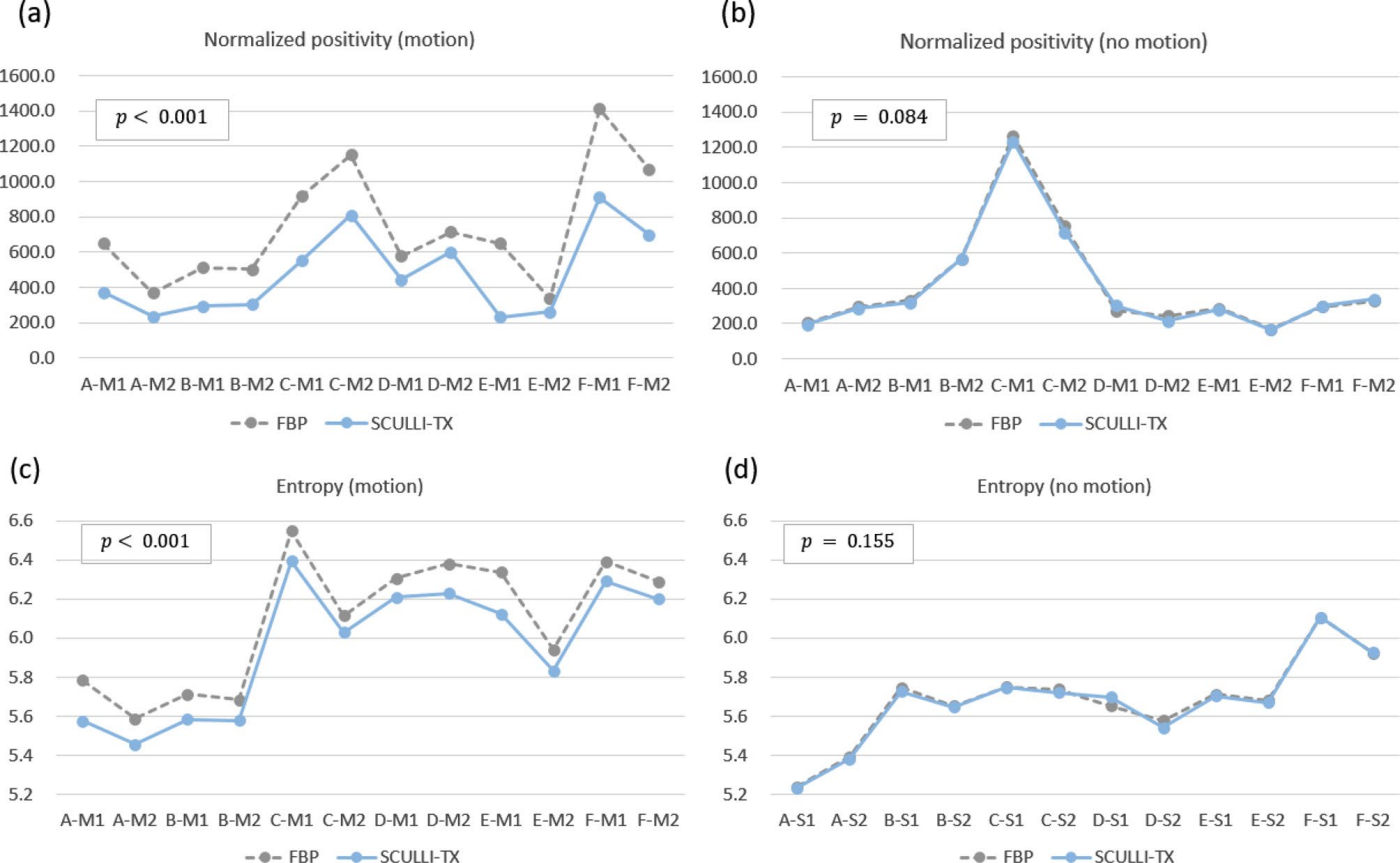
Results of quantitative evaluation using normalized positivity (top) and entropy (bottom). (a) SCULLI-TX provides significantly lower values of normalized positivity at ROIs of motion compared to FBP. (b) No significant difference of normalized positivity values observed between FBP and SCULLI-TX at ROIs of no motion. The same tendency for the entropy at the ROIs of (c) motion and (d) no motion respectively.

The results of quantitative assessment also show a consistent performance of SCULLI-TX as given in Fig. 6. There was a significant improvement by SCULLI-TX for the cases of motion artifacts. On average, we had 35.5% lower NP value (FBP: 738.3, SCULLI-TX: 476.1, $\mathrm{p}<0.001$) and 2.1% lower entropy value (FBP: 6.0885, SCULLI-TX: 5.9580, $\mathrm{p}<0.001$) of SCULLI-TX compared to those of FBP. In the images of FBP in Fig. 3, we can identify some representative appearances of motion artifacts in lung, which contributed to the larger values of the NP and entropy. First, there are seagull or star-like artifacts of lung parenchyma or lung vessels in Case A, B, C, D, E, and F. Second, lower intensities of CT-value or shadings near the lung structures were also prominent in Case A, B, C, E, and F. Lastly, the distortion of the airways were also noticeable as in Case B, C, D, E. These are consistent with the artifacts described in^1,2^ and these have been nicely alleviated by SCULLI-TX. In case E, some residual artifacts are observed near the heart. This indicates a potential limitation of SCULLI-TX at the heart borders against the adjacent lung segments under 0.5 s rotation and non-gating helical scans (with pitch 1.0). We address this more in Discussion. At the ROIs with no motion, there was no significant difference of NP (FBP: 418.0, SCULLI-TX: 409.8, $\mathrm{p}=0.084$) nor entropy (FBP: 5.6808, SCULLI-TX: 5.6748, $\mathrm{p}=0.155$) values. The images in Fig. 4 show no prominent difference between FBP and SCULLI-TX. In Supplementary Information (Figs. S2–S49) we provide the transaxial images of FBP and SCULLI-TX of motion and no motion, respectively.

**Figure 7:**
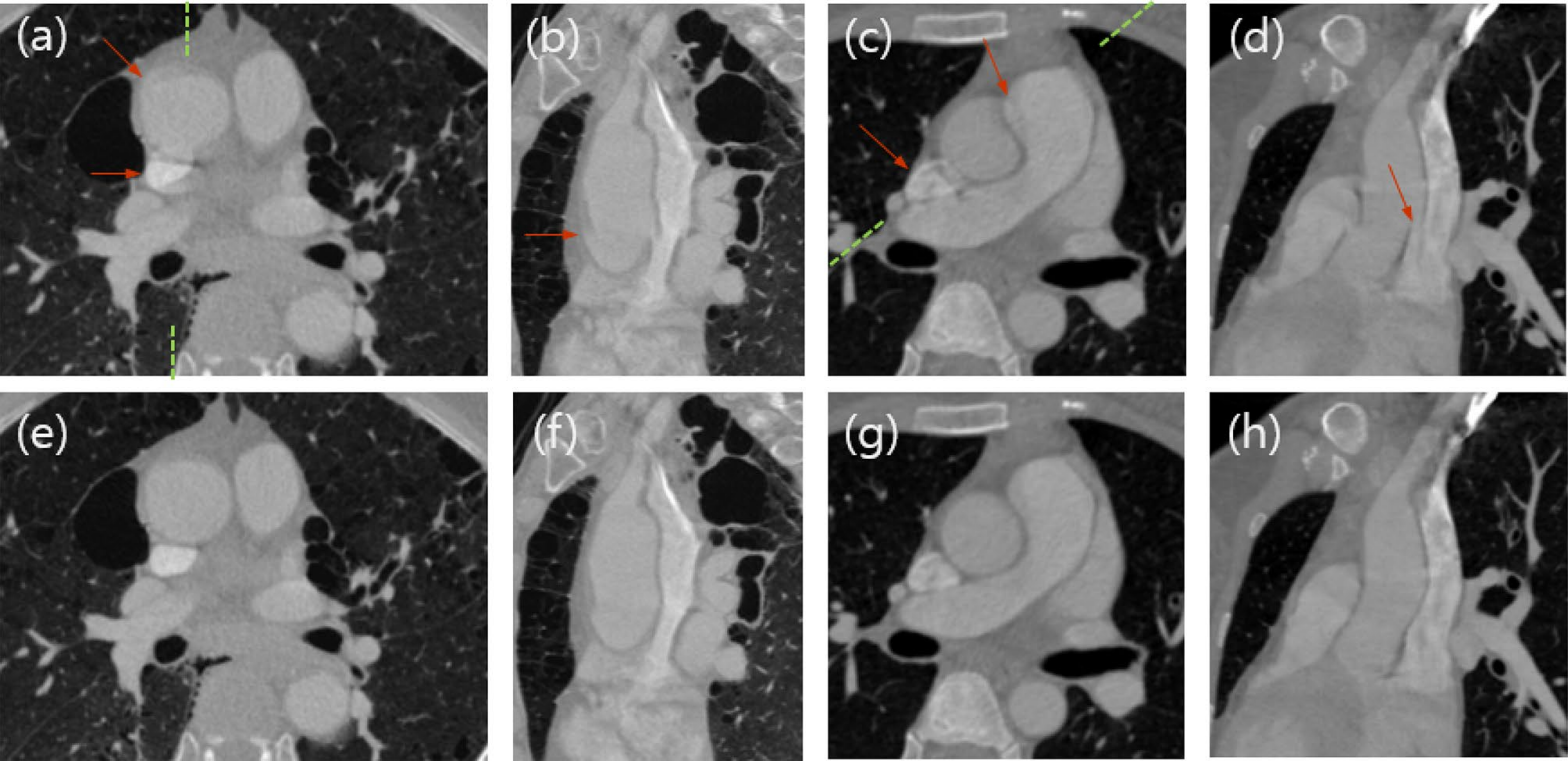
Images of FBP (top) and SCULLI-TX (bottom) of clinical case E and G. (a) Transaxial, (b) multi-planar images along the dotted green line of case E. Arrows indicate the motion artifacts of the aorta and the SVC that are corrected by SCULLI-TX in (e) and (f). L/W: − 400/2000 HU. (c), (d) Pseudo-flaps of the ascending aorta in transaxial and multi-planar images are observed in FBP of clinical case G. These have been removed by SCULLI-TX in (g) and (h). L/W: − 100/2000 HU.

SCULLI-TX showed a good potential in motion correction of thoracic structures as well. The FBP images of case E and G show a typical pseudo-flaps of the ascending aorta mimicking aortic dissection in Fig. 7(a)–(d). The neighboring SVC also suffers from motion artifacts. The multi-planar images show the corresponding z-location at the ascending aorta which is pulsating together with the heart. SCULLI-TX visualizes the delineation of the ascending aorta and the SVC nicely in Fig. 7(e)–(h).

## Discussion

There are some limitations in this study. First, the motion of the COPD phantom attached to QSP actuator was in-plane translation. In addition, the motion of the COPD phantom was rigid, which is different from the elastic motion of the lung structures. As described as color mapping in^3^, the transmitted motion of the lung structures is inversely proportional to the distance from the heart. More sophisticated phantom studies considering three dimensional elastic motion of lung would be the next step. Second, we did not consider the heart rates of the human subjects. Since the motion of lung and thoracic structures are directly related to the heartbeat or heart motion^19^, it would be very meaningful to investigate the performance of SCULLI-TX accordingly. Third, we did not perform quantification of lung exams in commercially available clinical workstations, such as airway wall thickness, volume of nodule etc. As we observed in the results of the clinical cases, the lung structures are very sensitive to motion artifacts, which could lead to errors in the quantification task^6^.

In this study we demonstrated that the SCULLI-TX provides a significant improvement of image quality by reducing motion artifacts. However, it remains unknown whether it can change the diagnosis. Investigating the diagnostic benefits achieved by SCULLI-TX would be the next step. Although the two measures showed a good match with the qualitative evaluation of the authors regarding the existence of the motion artifacts, they are not absolute measure but relative. In addition, two measures showed a different scale of improvement in case of motion. For these reasons, it is a very interesting research topic to explore an absolute measure which can quantify the various appearance of the lung motion artifacts. For example, convolutional neural network (CNN) could be adopted for motion quantification^20^.

The results demonstrated in this study support a great potential of the proposed method in various applications. Since it does not require a dedicated acquisition mode, but works for routine CT scan modes such as axial and helical scan with pitch not larger than 1.0. For instance, SCULLI-TX can be adopted for rule-out of aortic dissection in emergency department^18,19^. Pediatric chest CT exams^21^ or calcium scoring under low dose lung screening^22^ are also important applications for which the removal of motion artifacts is crucial. Since low dose scan protocols are generally utilized in the exams, we can combine SCULLI-TX with noise reduction techniques^23^. In case of calcium scoring, we can also consider more sophisticated motion estimation^24^ to deal with the motion of the coronary arteries from non-gating chest scans with ordinary helical pitch. Lung quantification^6^ is also a potential application where SCULLI-TX can come into play in improving temporal resolution. Lastly but not least, it can be adopted for radiation therapy planning. Here, we do not know yet whether SCULLI-TX could correct the breathing motion too. So far, we only showed the potential^9^ using XCAT simulation study in this regard. We are looking forward to exploring the capability of the proposed method in the various clinical applications.

## Supplementary Information

Supplementary Information.

## Data Availability

All data generated or analyzed during this study are included in this published article (and its Supplementary Information files).
