# Supplementary material for: Motion correction for routine X-ray lung CT imaging
Source: Sci Rep. 2021 Feb 12;11:3695. doi: 10.1038/s41598-021-83403-w (PMC7880999; doi:10.1038/s41598-021-83403-w)
Supplement: Supplementary file 1 — Supplementary Information. [file 41598_2021_83403_MOESM1_ESM.pdf]

Motion Correction For Routine X-ray Lung CT Imaging

Doil Kim[1], Jiyoung Choi[1], Duhgoon Lee[1], Hyesun Kim[1], Jiyoung Jung[1], Minkook Cho[1], and Kyoung-Yong Lee[1,*]

[1]CT R&D Group, Health & Medical Equipment Business, Samsung Electronics Co., Ltd., Suwon, Republic of Korea

[*]Corresponding author. E-mail: ky71.lee@samsung.com

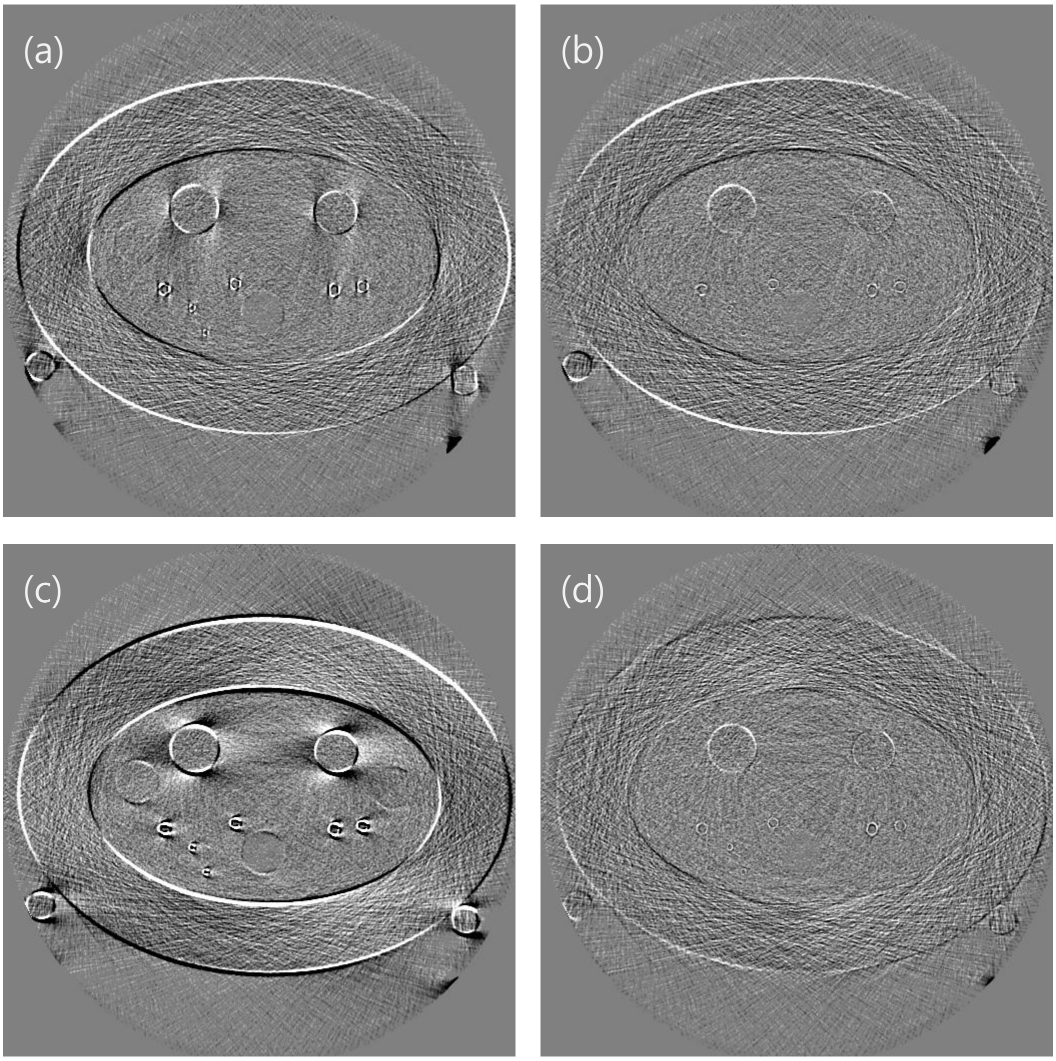

Supplementary Figure S1. Difference images between the ground truth and (a) 10 rpm FBP (b) 10 rpm SCULLI-TX (c) 15 rpm FBP (d) 15 rpm SCULLI-TX, respectively. (L/W: 0/200 HU, magnification: 50%) Errors due to positioning mismatch, distortion of the structures, streaks and shadings arising as a result of motion are prominent in (a) and (c) which have been significantly reduced in (b) and (d). Some edges of the structures due to slightly different slice locations are visible in (b) and (d).

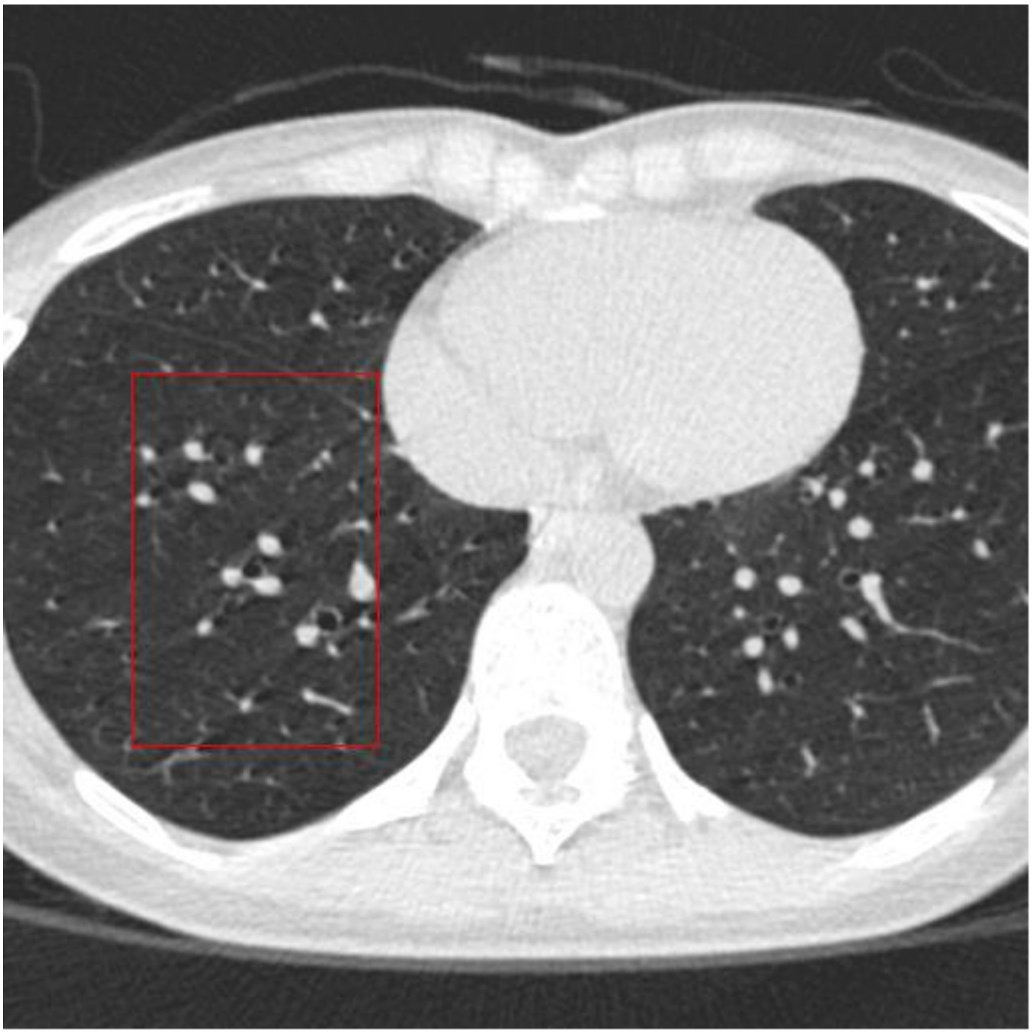

Supplementary Figure S2. FBP image of motion artifacts (Case A-M1). L/W: -500/1500 HU.

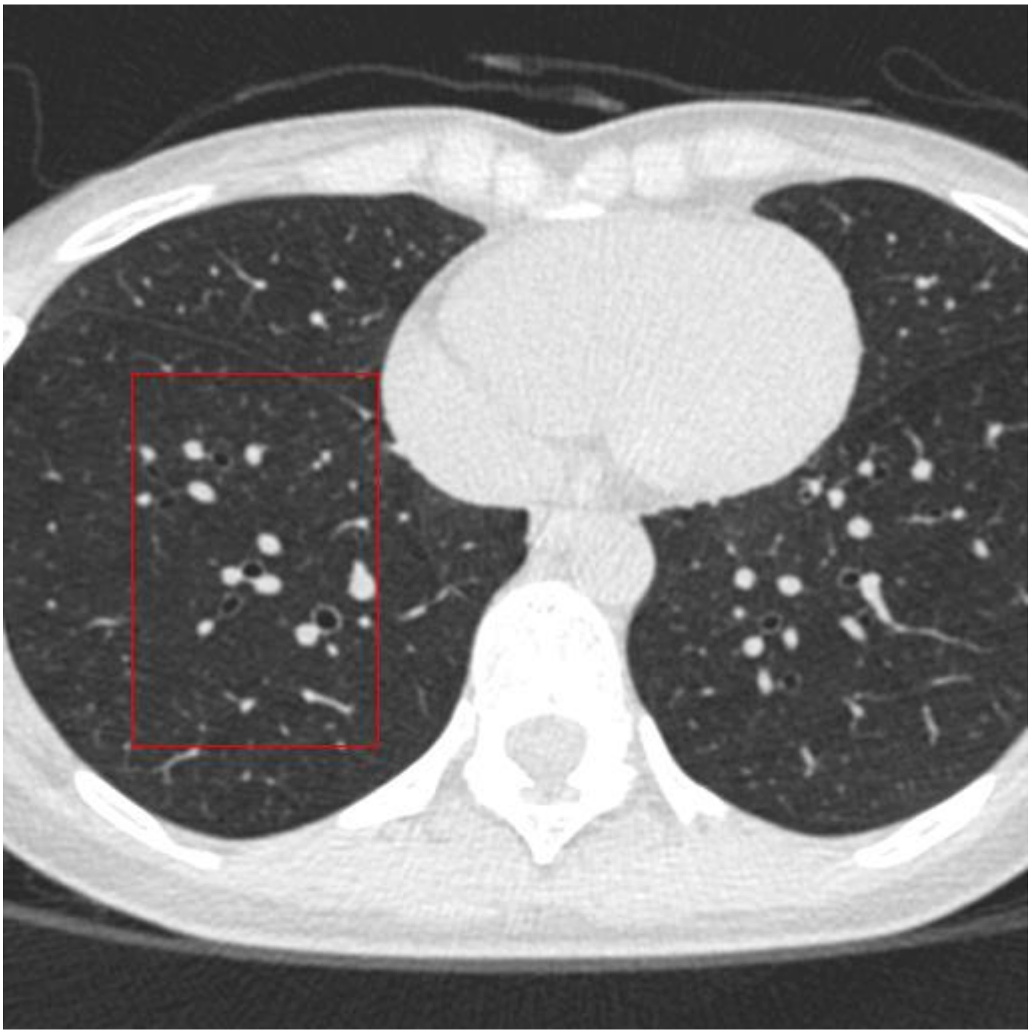

Supplementary Figure S3. SCULLI-TX image of Case A-M1. L/W: -500/1500 HU.

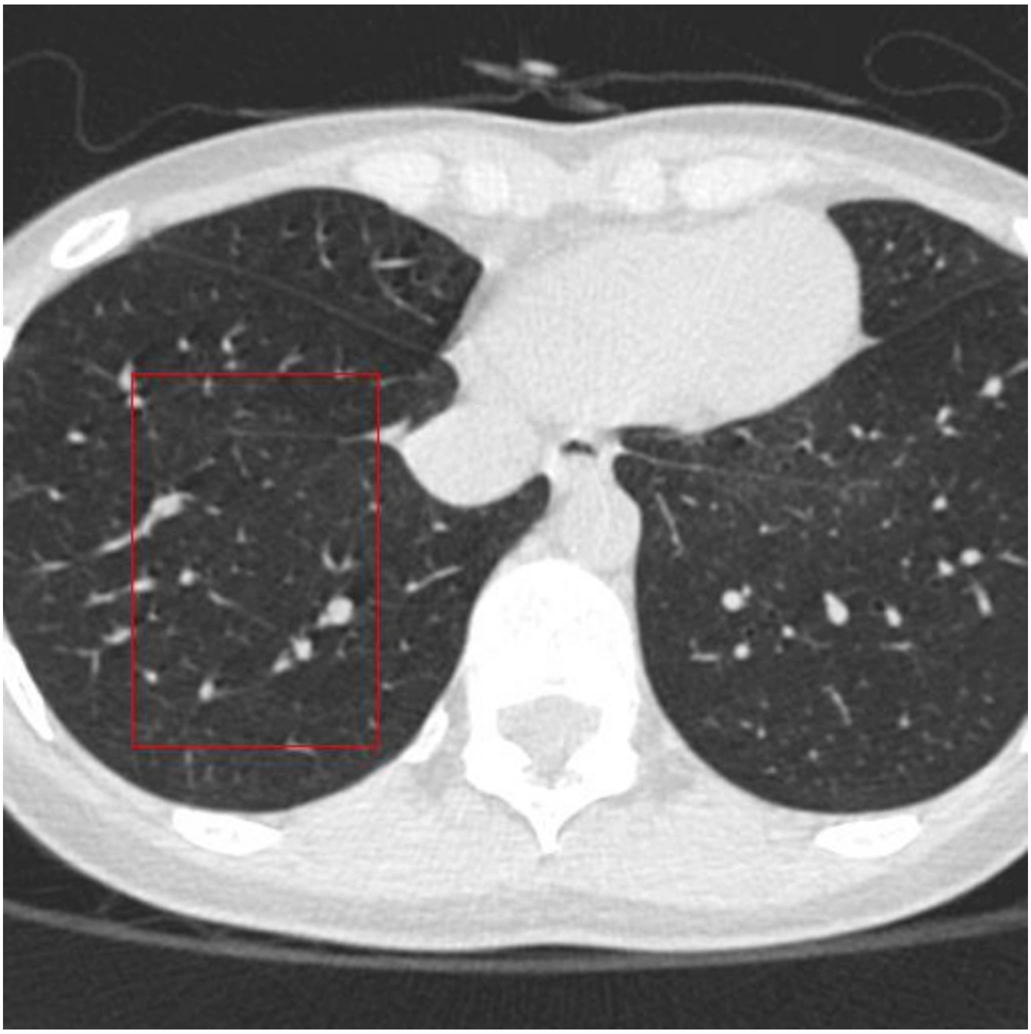

Supplementary Figure S4. FBP image of motion artifacts (Case A-M2). L/W: -500/1500 HU.

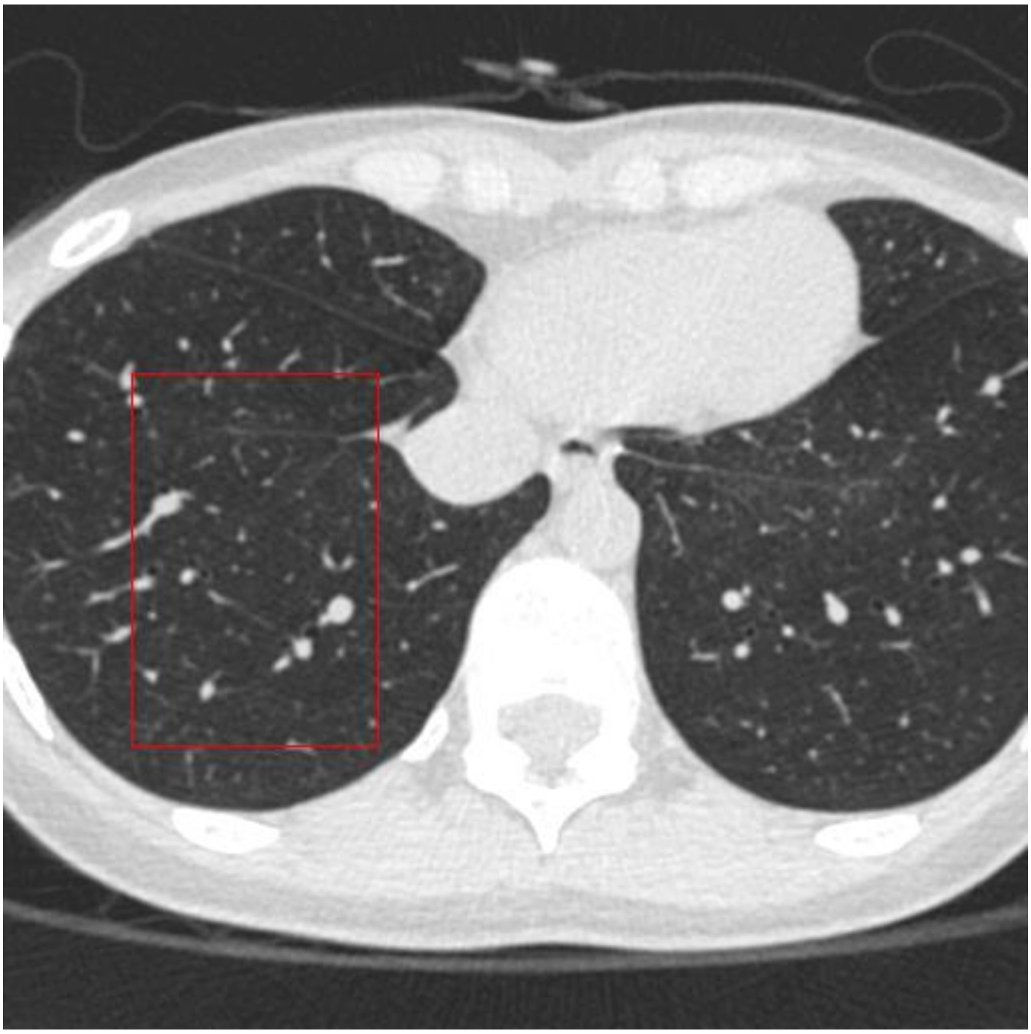

Supplementary Figure S5. SCULLI-TX image of Case A-M2. L/W: -500/1500 HU.

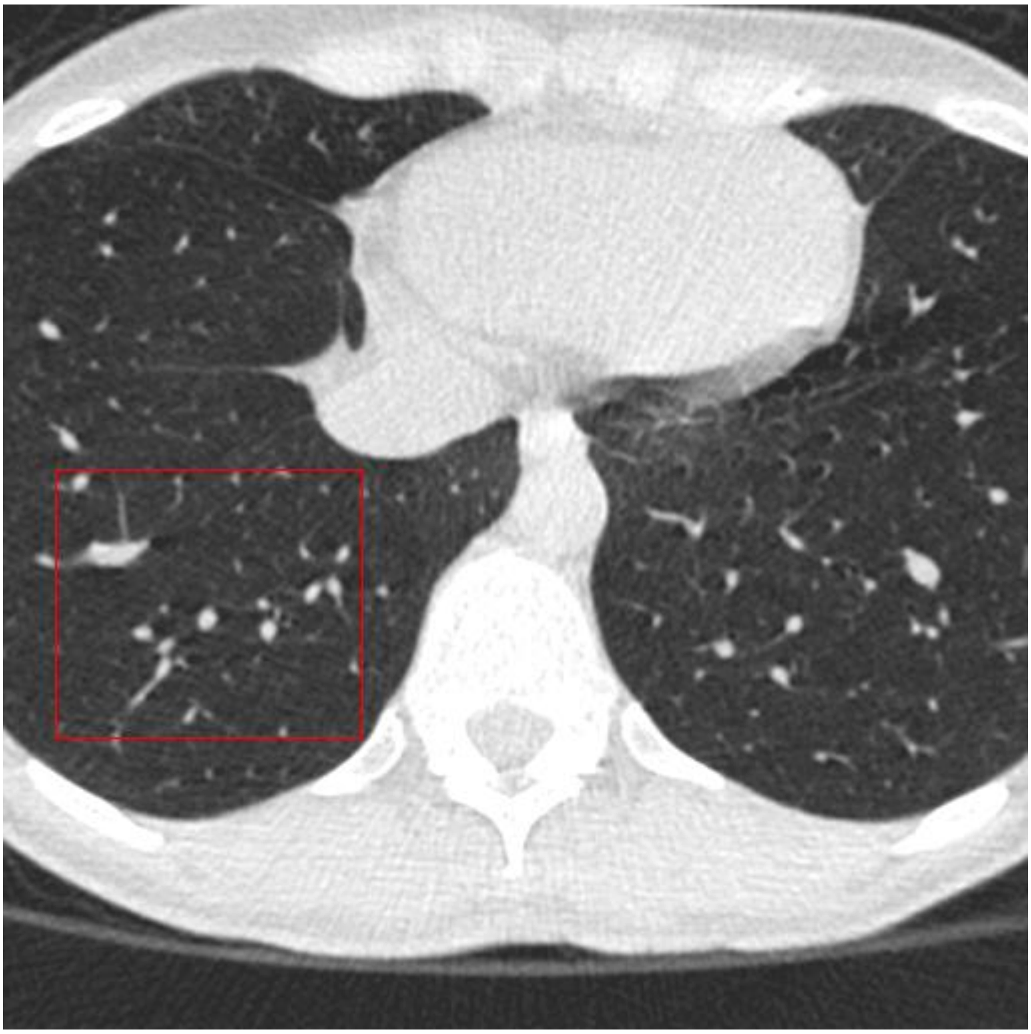

Supplementary Figure S6. FBP image of motion artifacts (Case B-M1). L/W: -500/1500 HU.

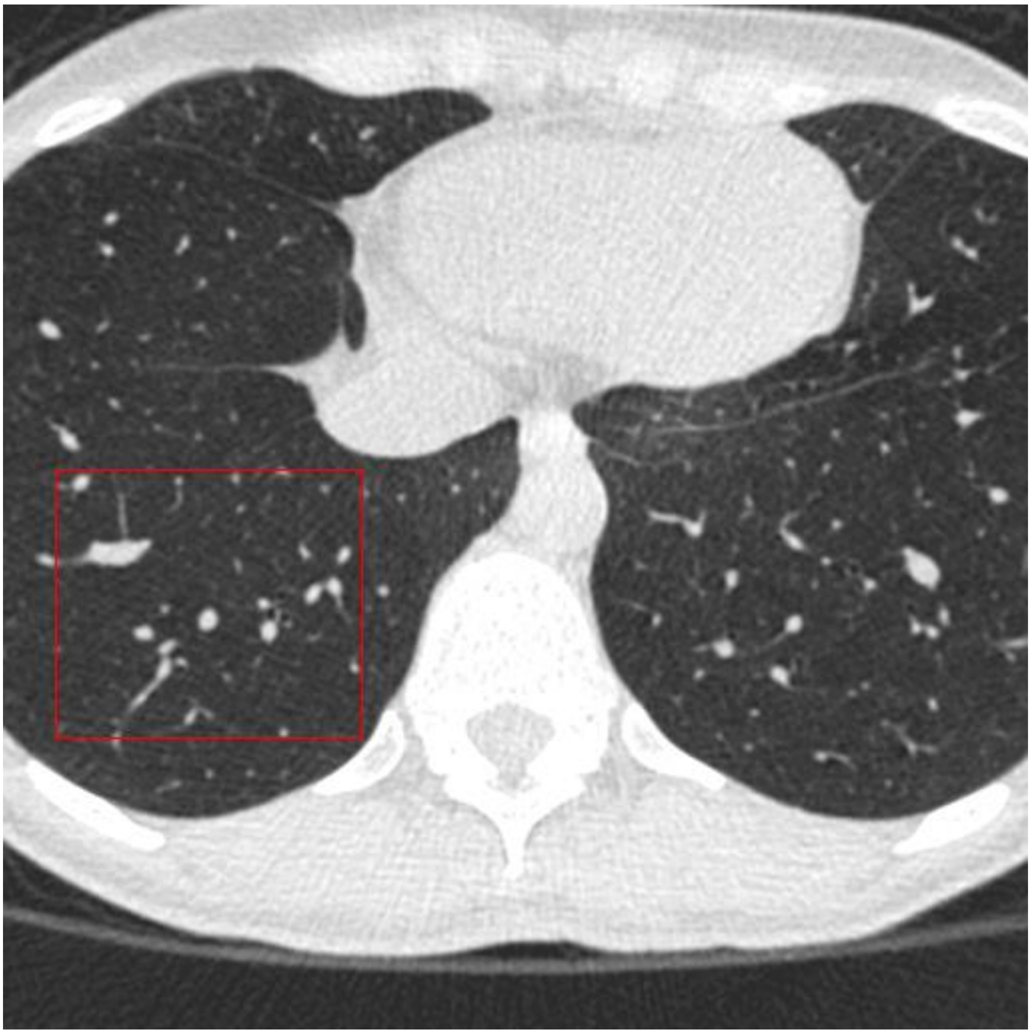

Supplementary Figure S7. SCULLI-TX image of Case B-M1. L/W: -500/1500 HU.

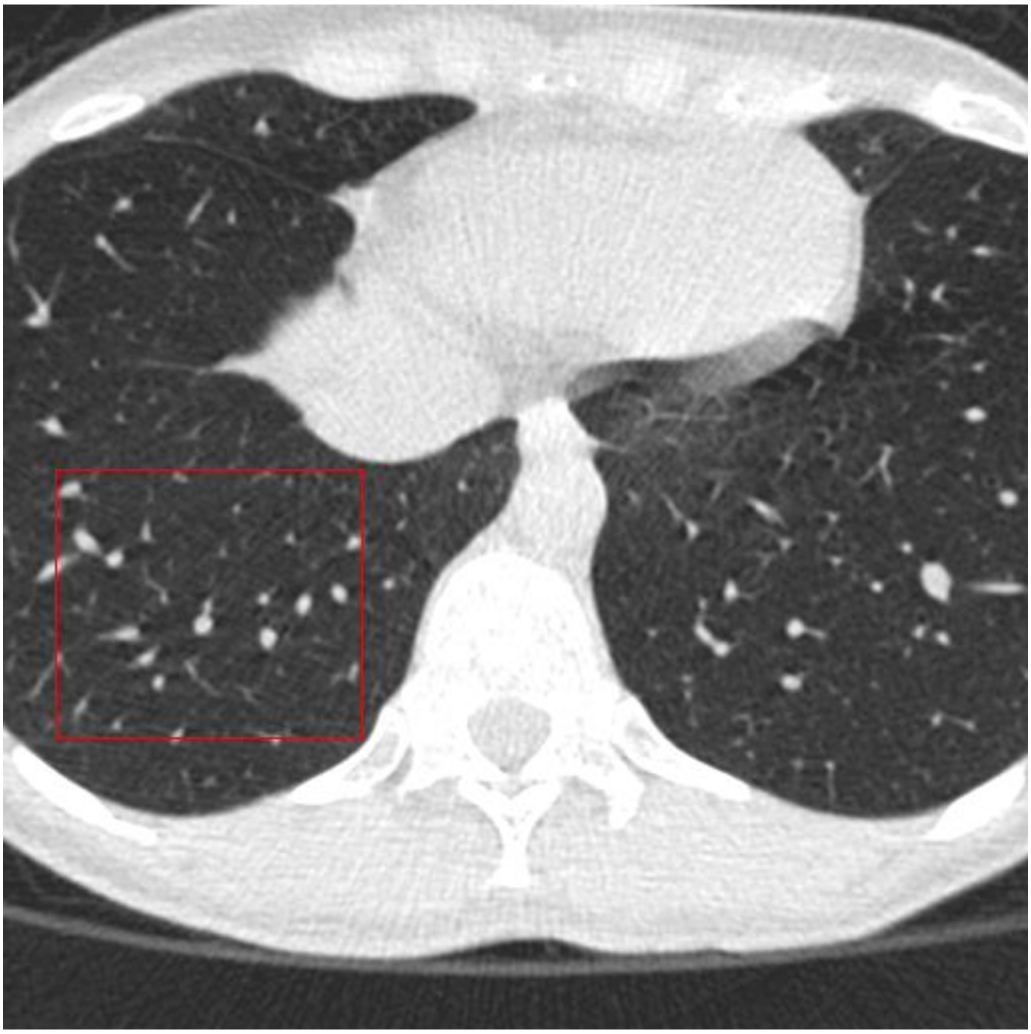

Supplementary Figure S8. FBP image of motion artifacts (Case B-M2). L/W: -500/1500 HU.

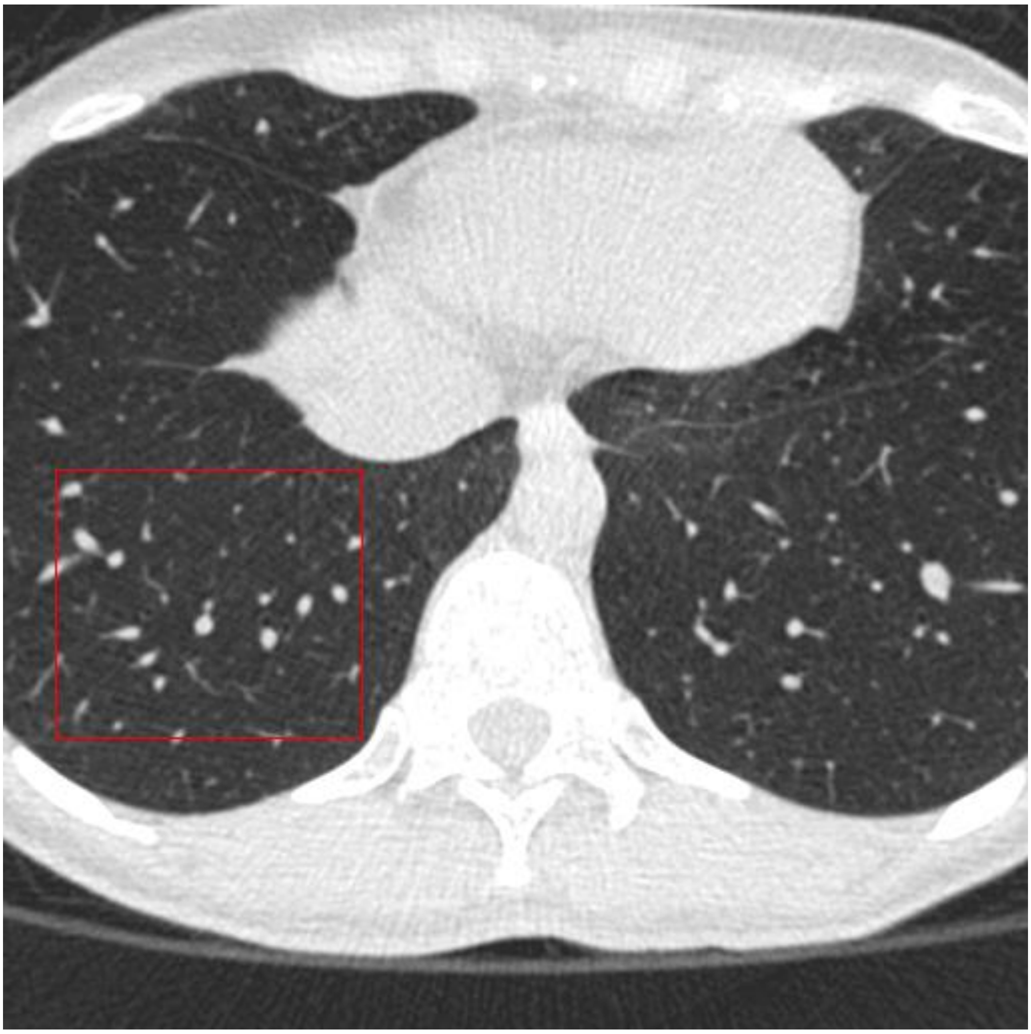

Supplementary Figure S9. SCULLI-TX image of Case B-M2. L/W: -500/1500 HU.

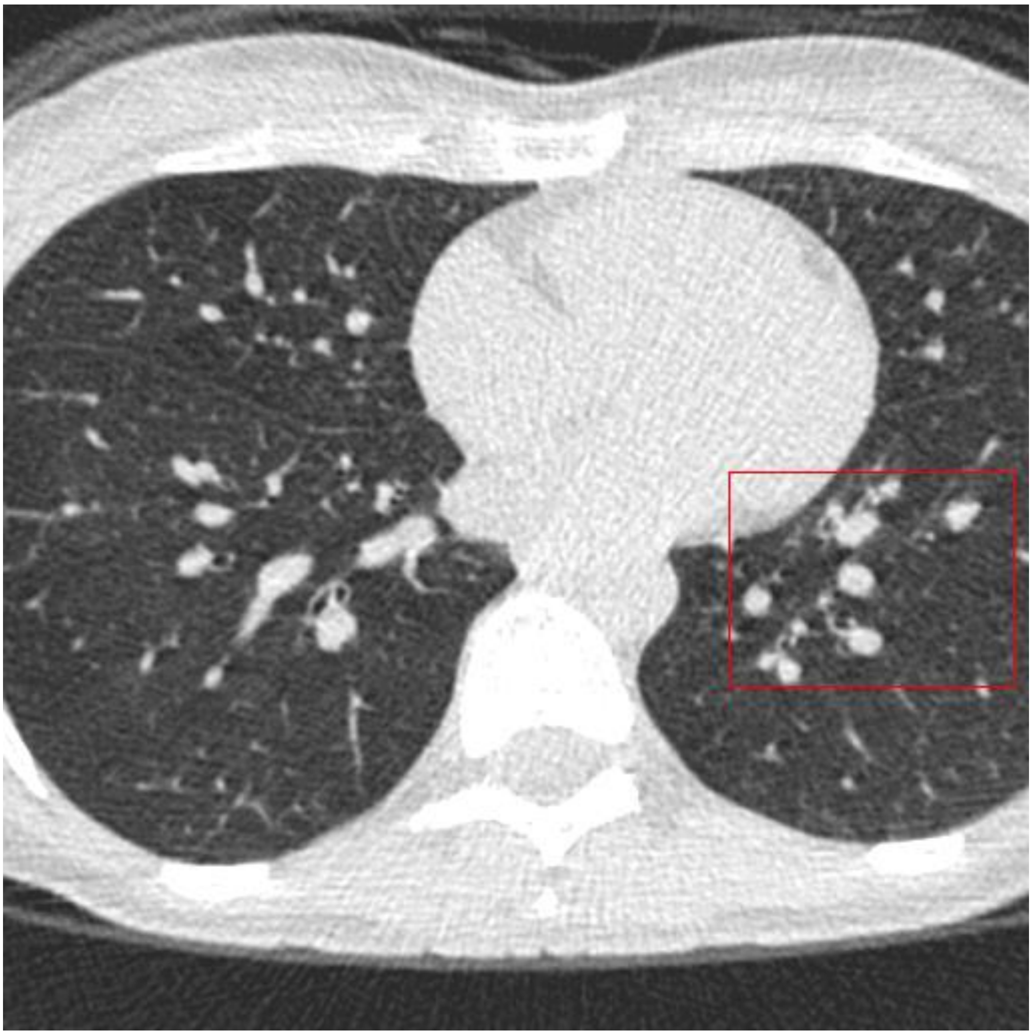

Supplementary Figure S10. FBP image of motion artifacts (Case C-M1). L/W: -500/1500 HU.

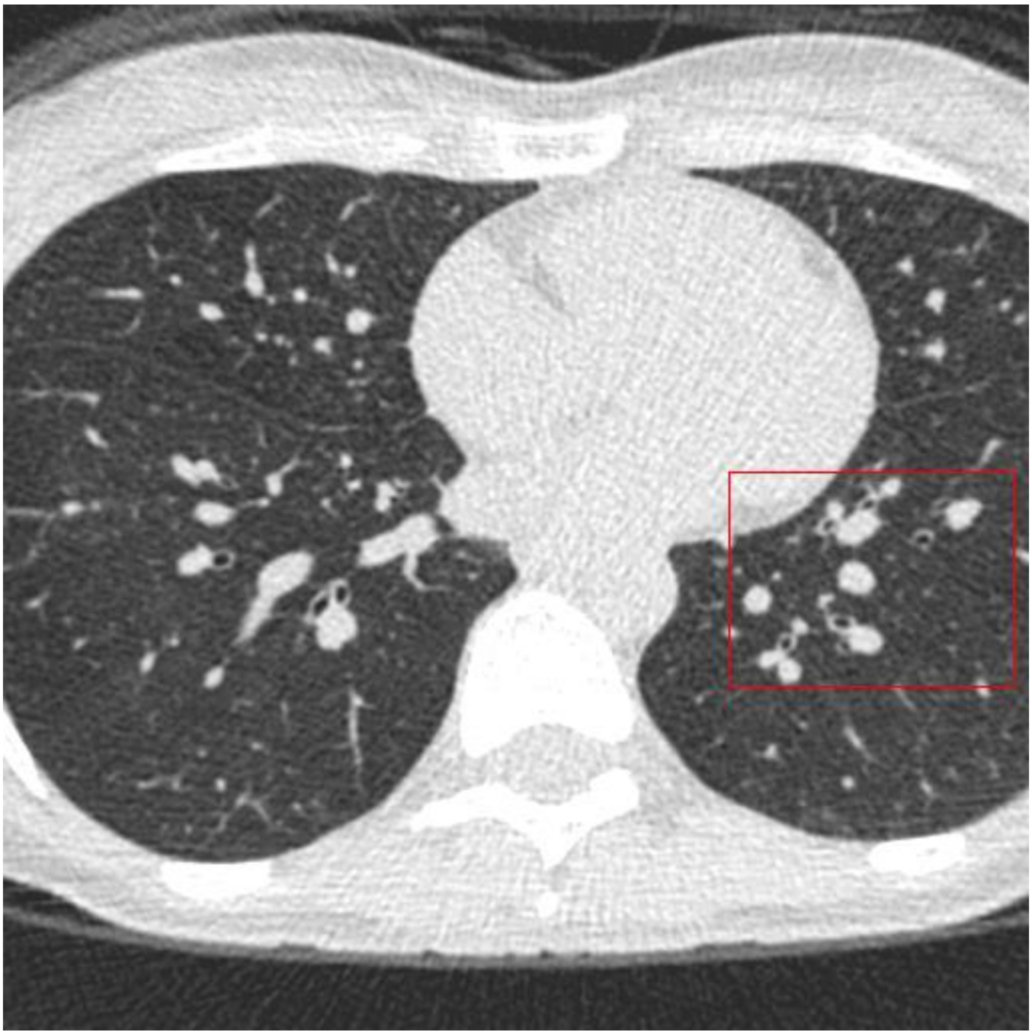

Supplementary Figure S11. SCULLI-TX image of Case C-M1. L/W: -500/1500 HU.

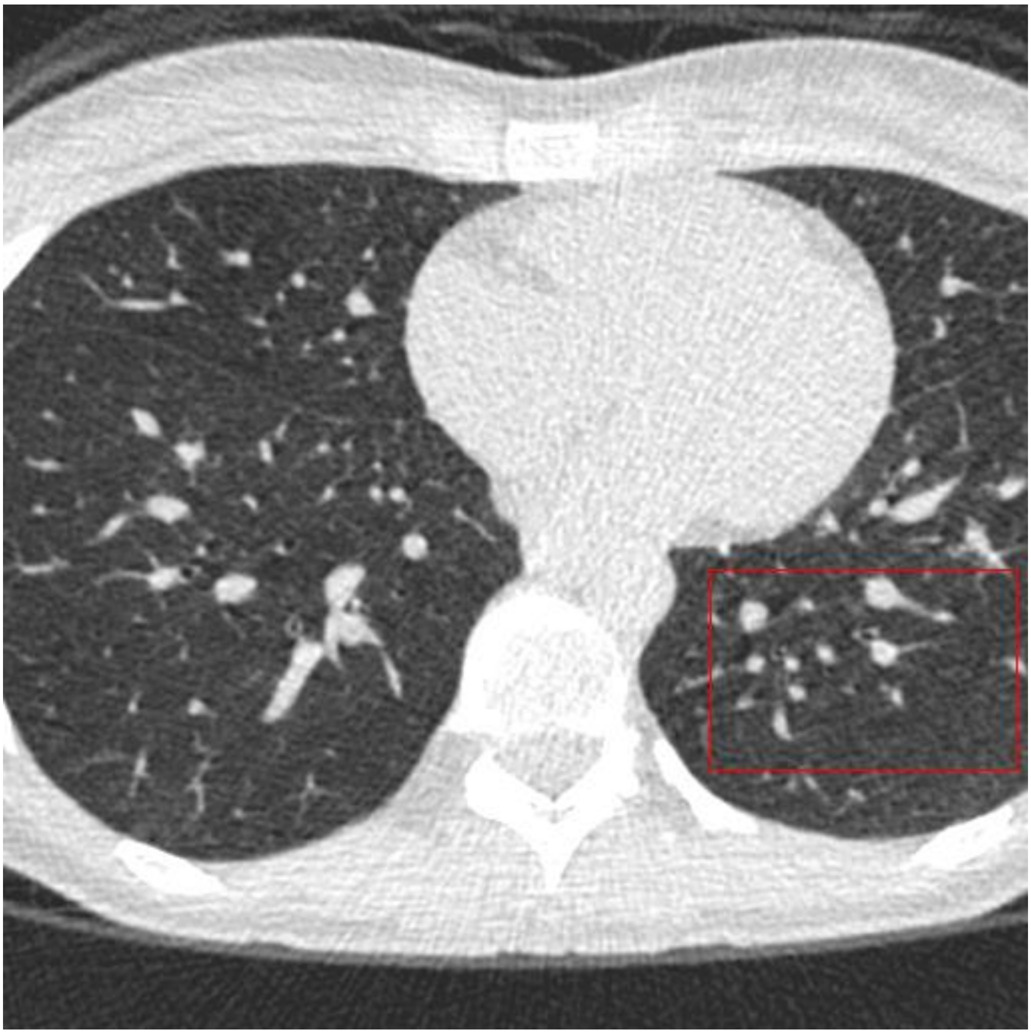

Supplementary Figure S12. FBP image of motion artifacts (Case C-M2). L/W: -500/1500 HU.

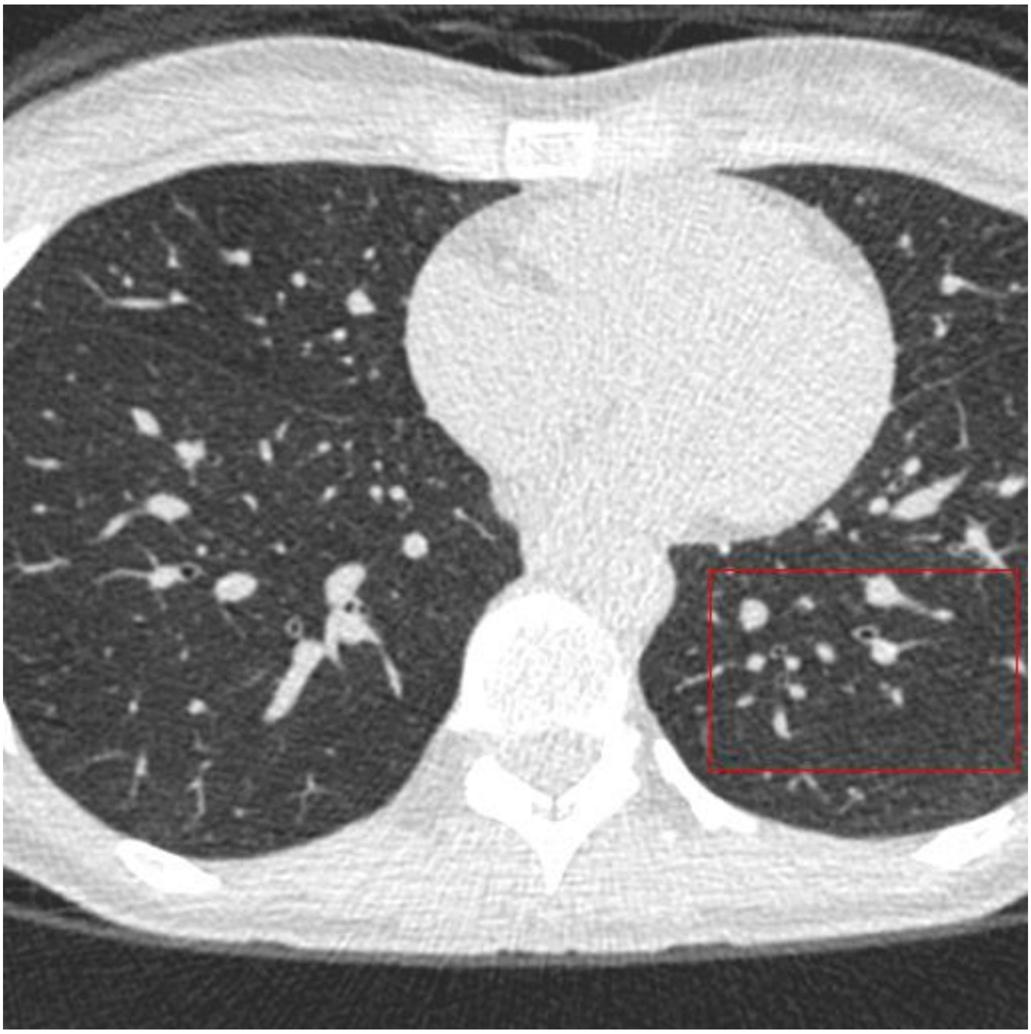

Supplementary Figure S13. SCULLI-TX image of Case C-M2. L/W: -500/1500 HU.

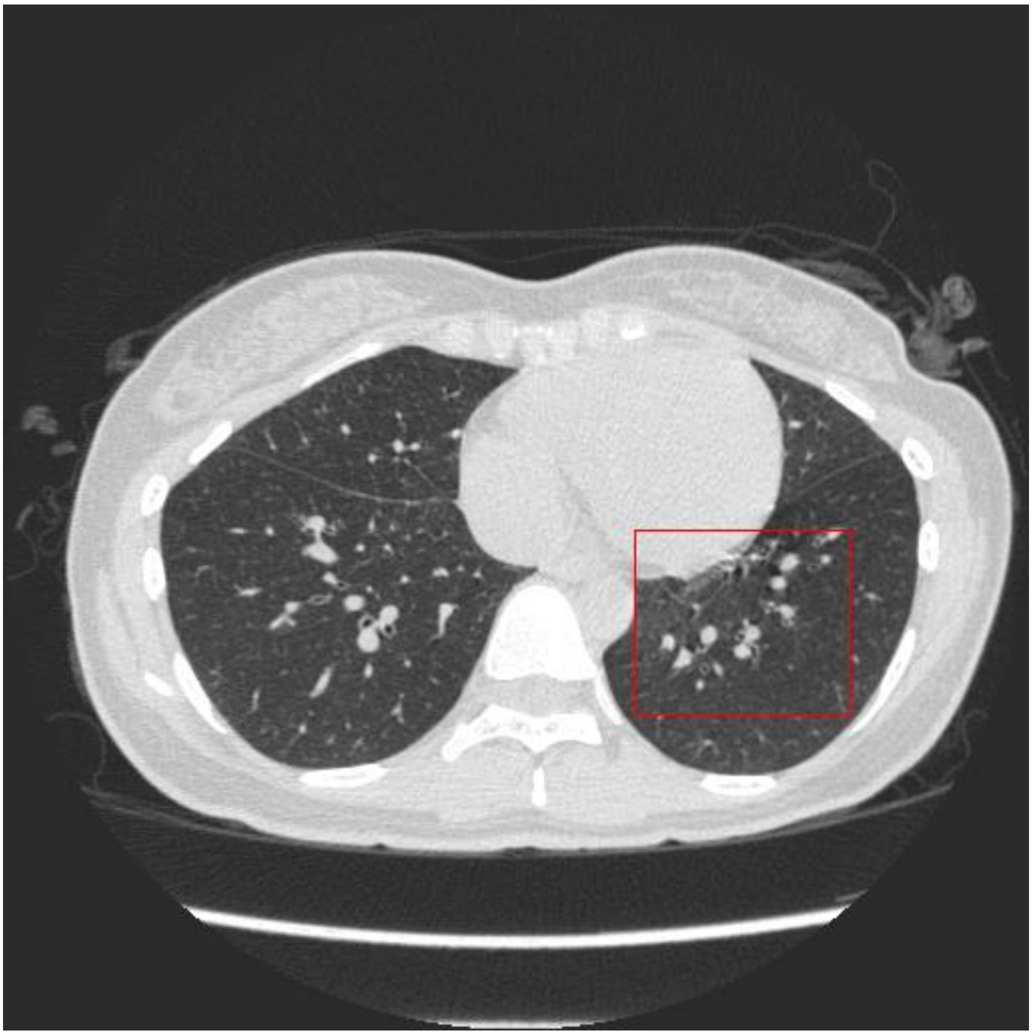

Supplementary Figure S14. FBP image of motion artifacts (Case D-M1). L/W: -500/1500 HU.

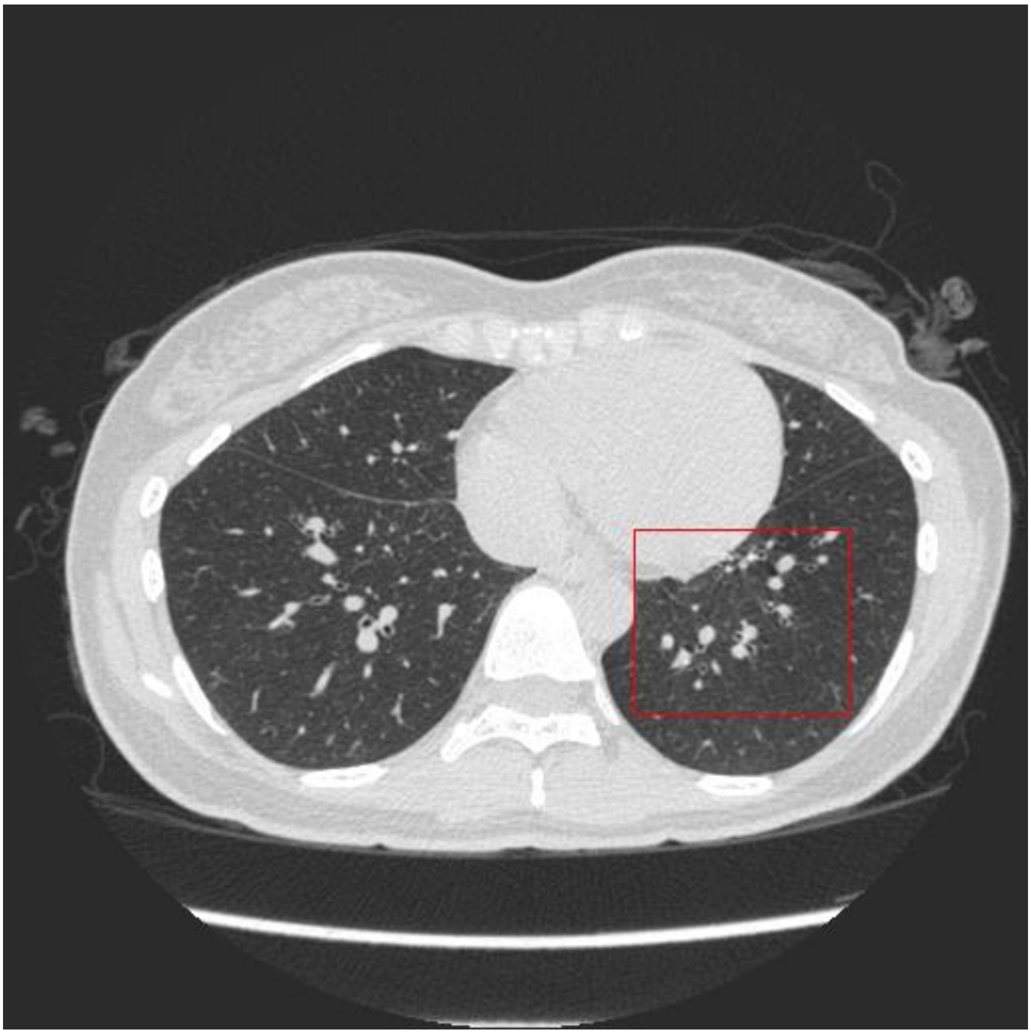

Supplementary Figure S15. SCULLI-TX image of Case D-M1. L/W: -500/1500 HU.

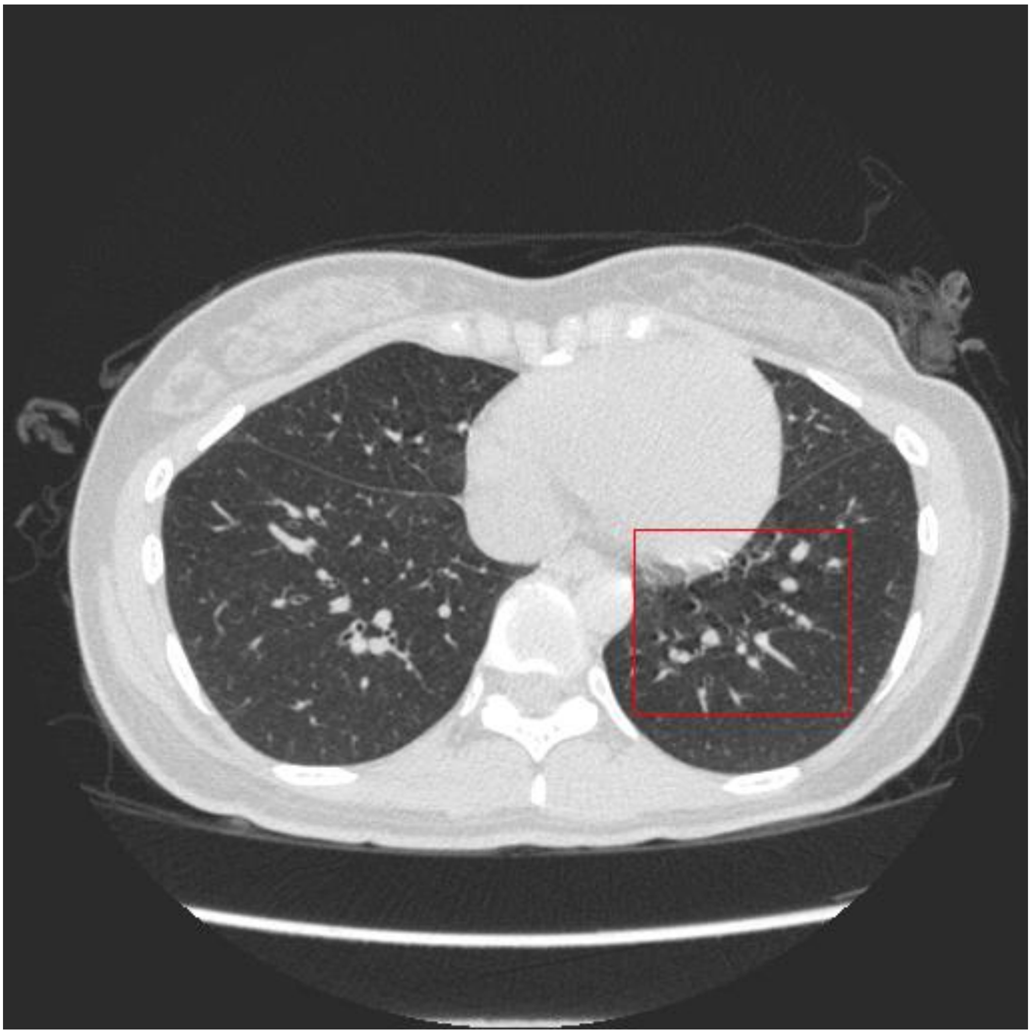

Supplementary Figure S16. FBP image of motion artifacts (Case D-M2). L/W: -500/1500 HU.

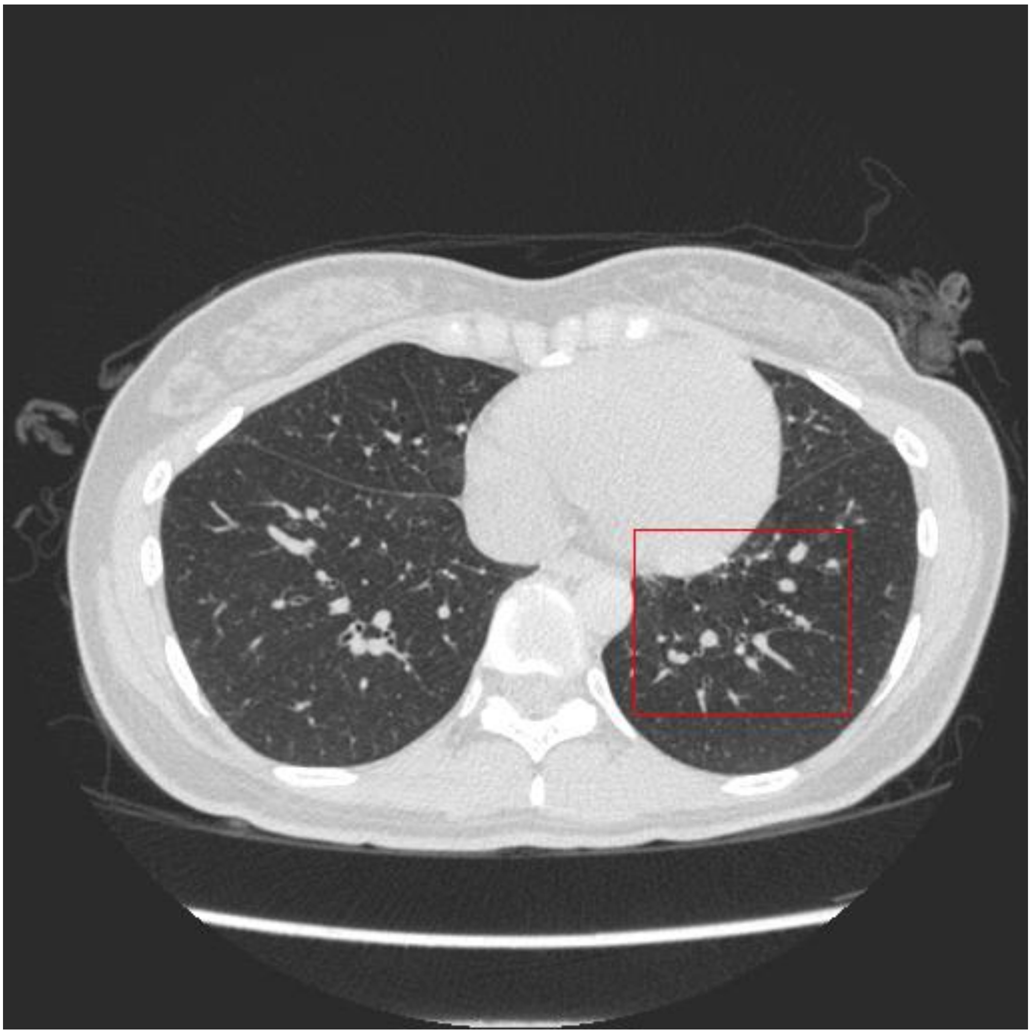

Supplementary Figure S17. SCULLI-TX image of Case D-M2. L/W: -500/1500 HU.

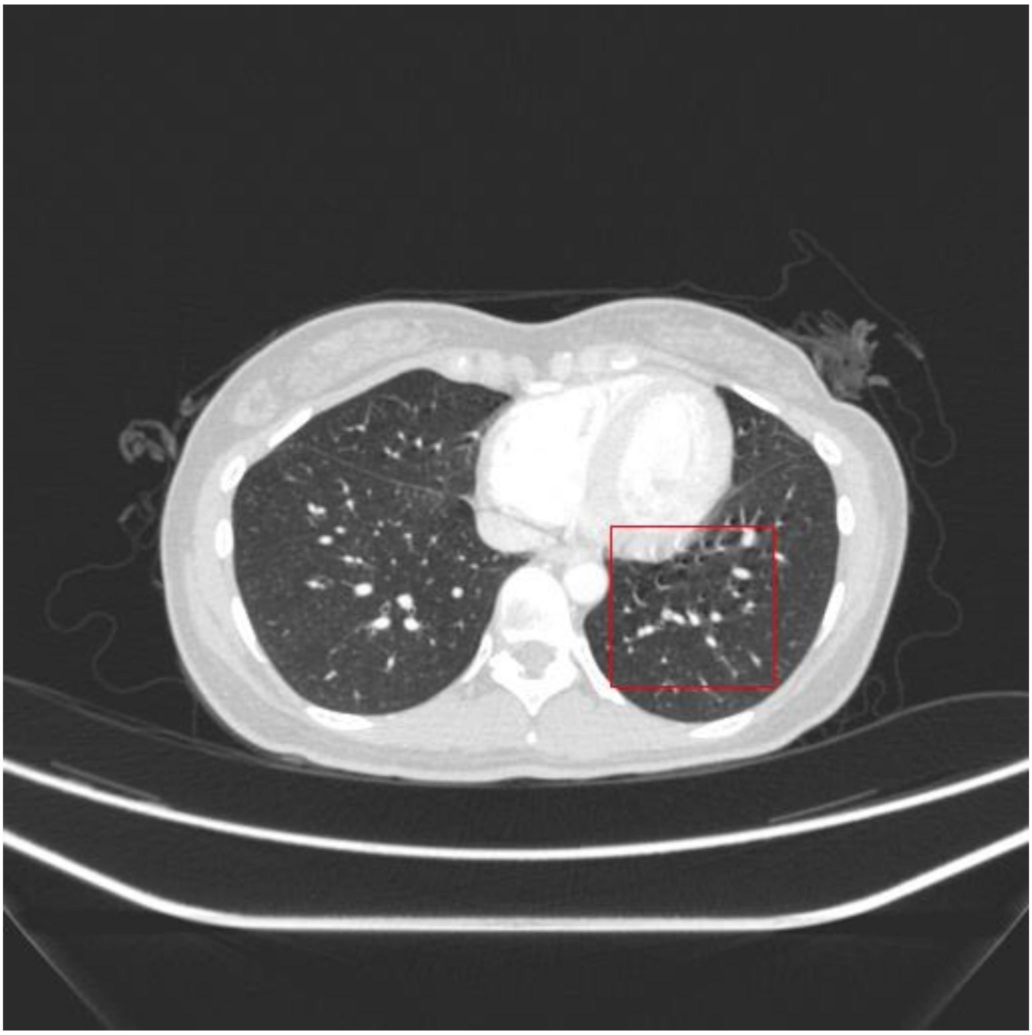

Supplementary Figure S18. FBP image of motion artifacts (Case E-M1). L/W: -500/1500 HU.

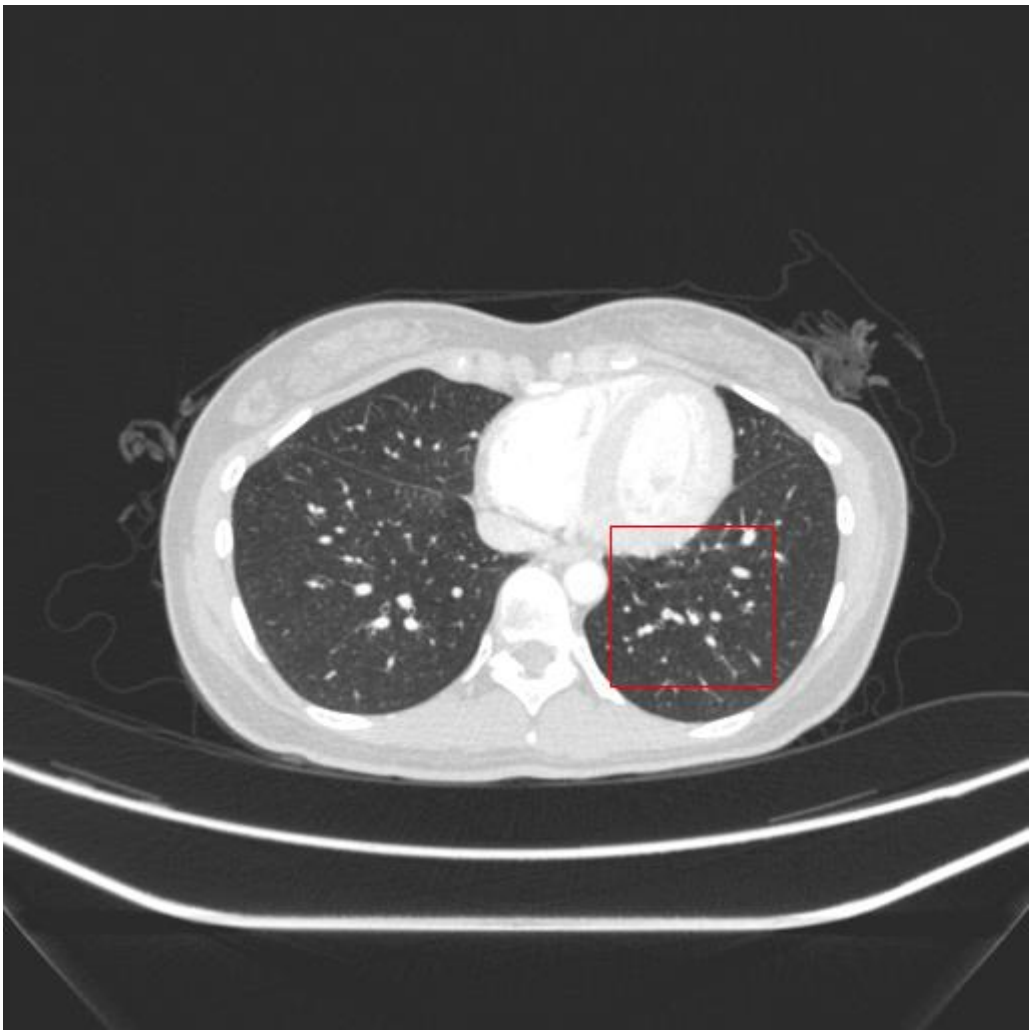

Supplementary Figure S19. SCULLI-TX image of Case E-M1. L/W: -500/1500 HU.

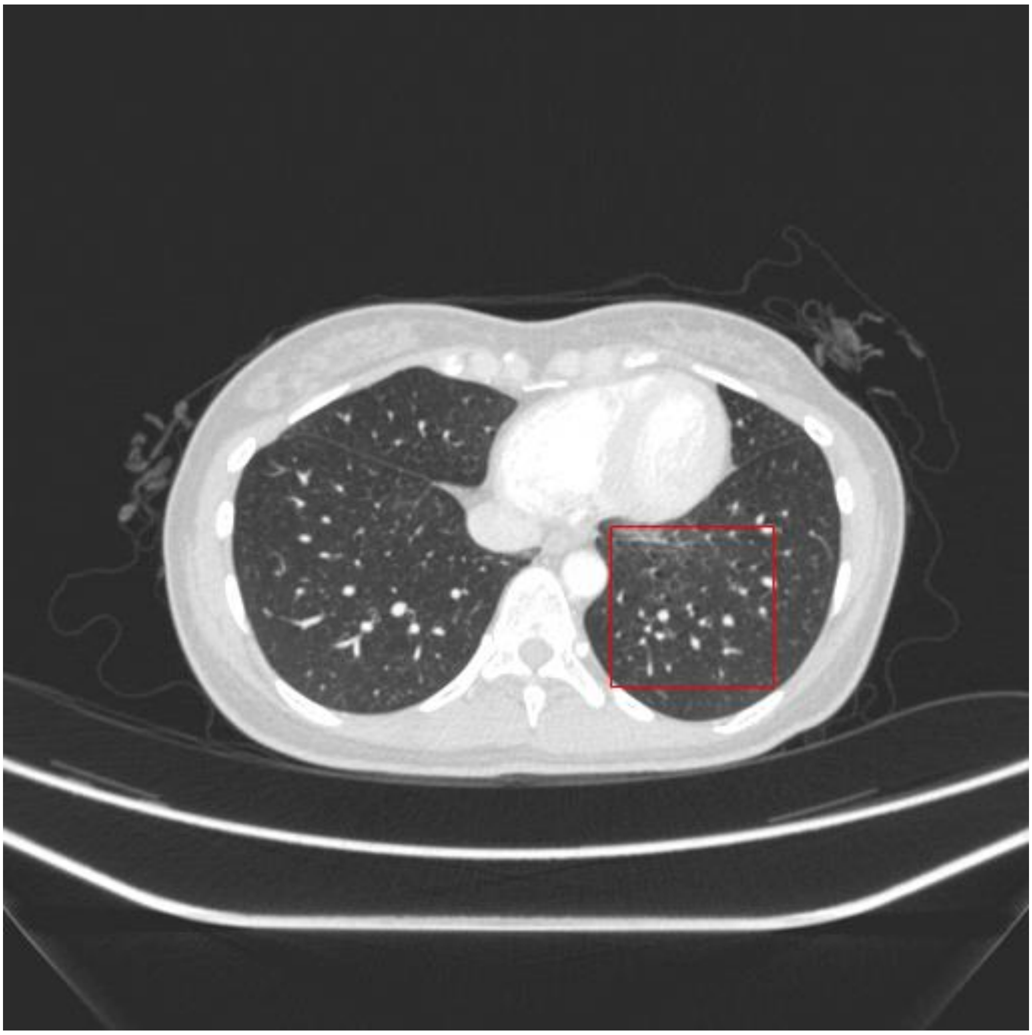

Supplementary Figure S20. FBP image of motion artifacts (Case E-M2). L/W: -500/1500 HU.

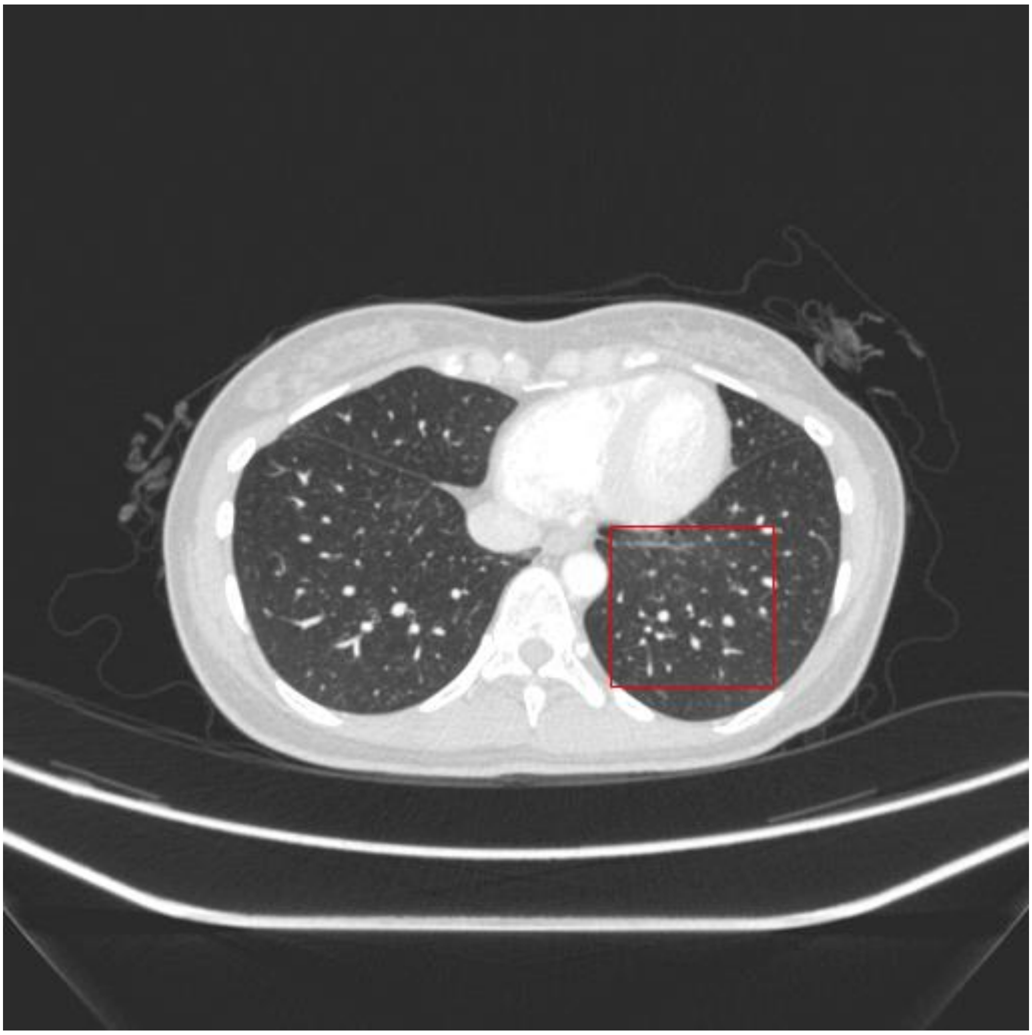

Supplementary Figure S21. SCULLI-TX image of Case E-M2. L/W: -500/1500 HU.

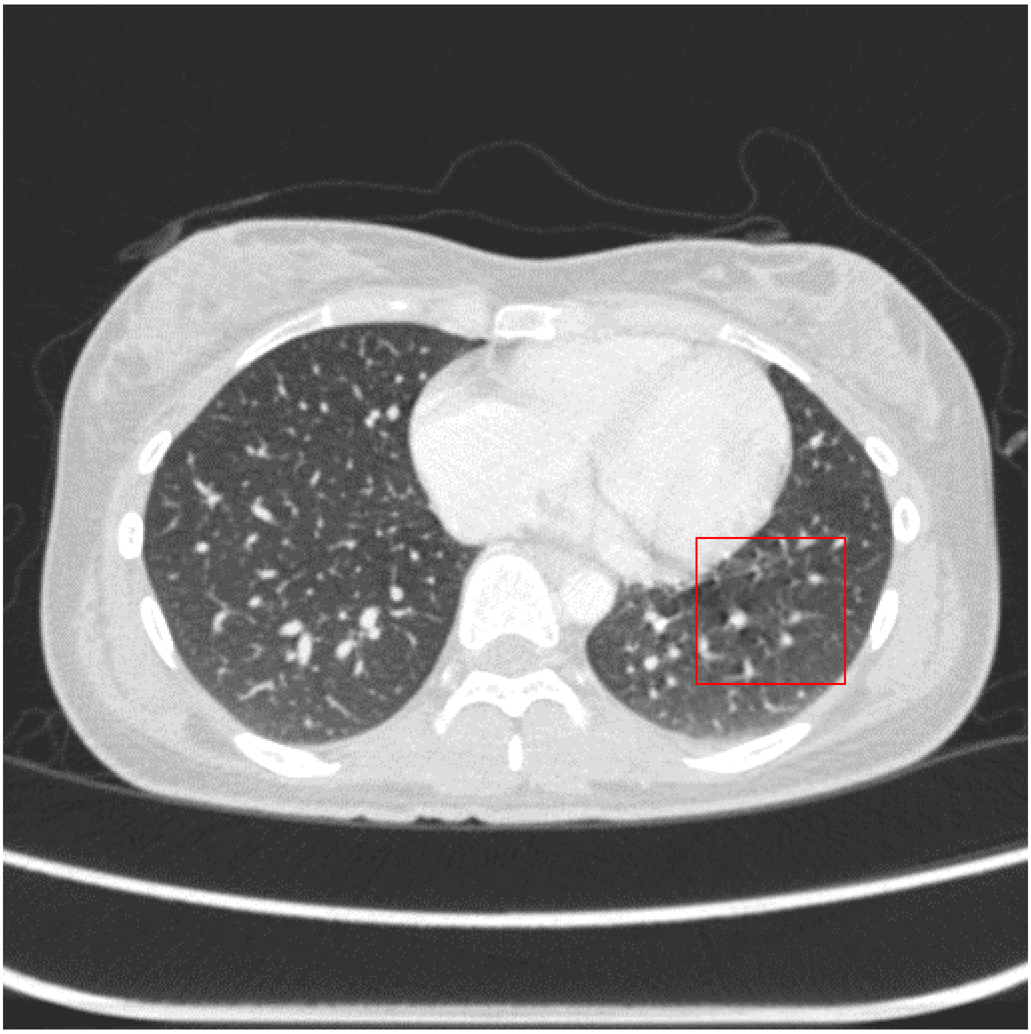

Supplementary Figure S22. FBP image of motion artifacts (Case F-M1). L/W: -500/1500 HU.

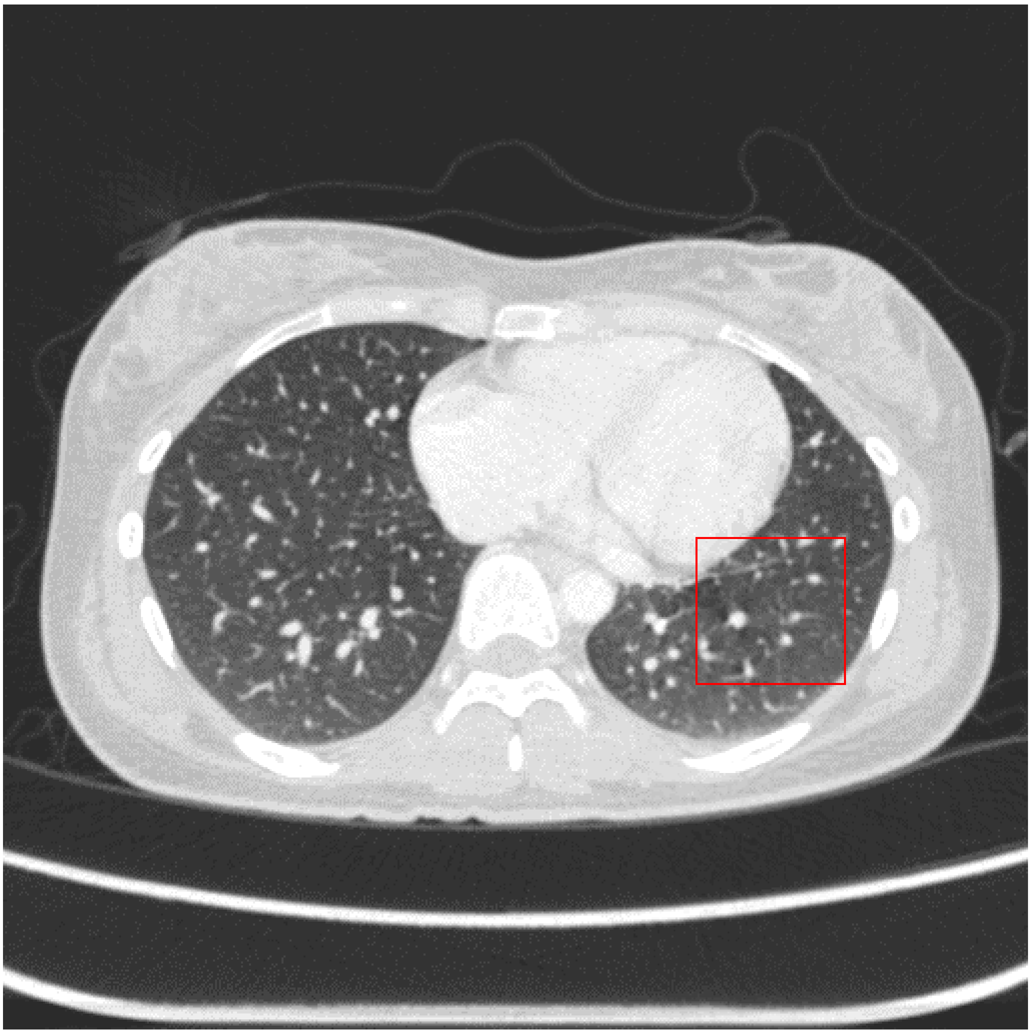

Supplementary Figure S23. SCULLI-TX image of Case F-M1. L/W: -500/1500 HU.

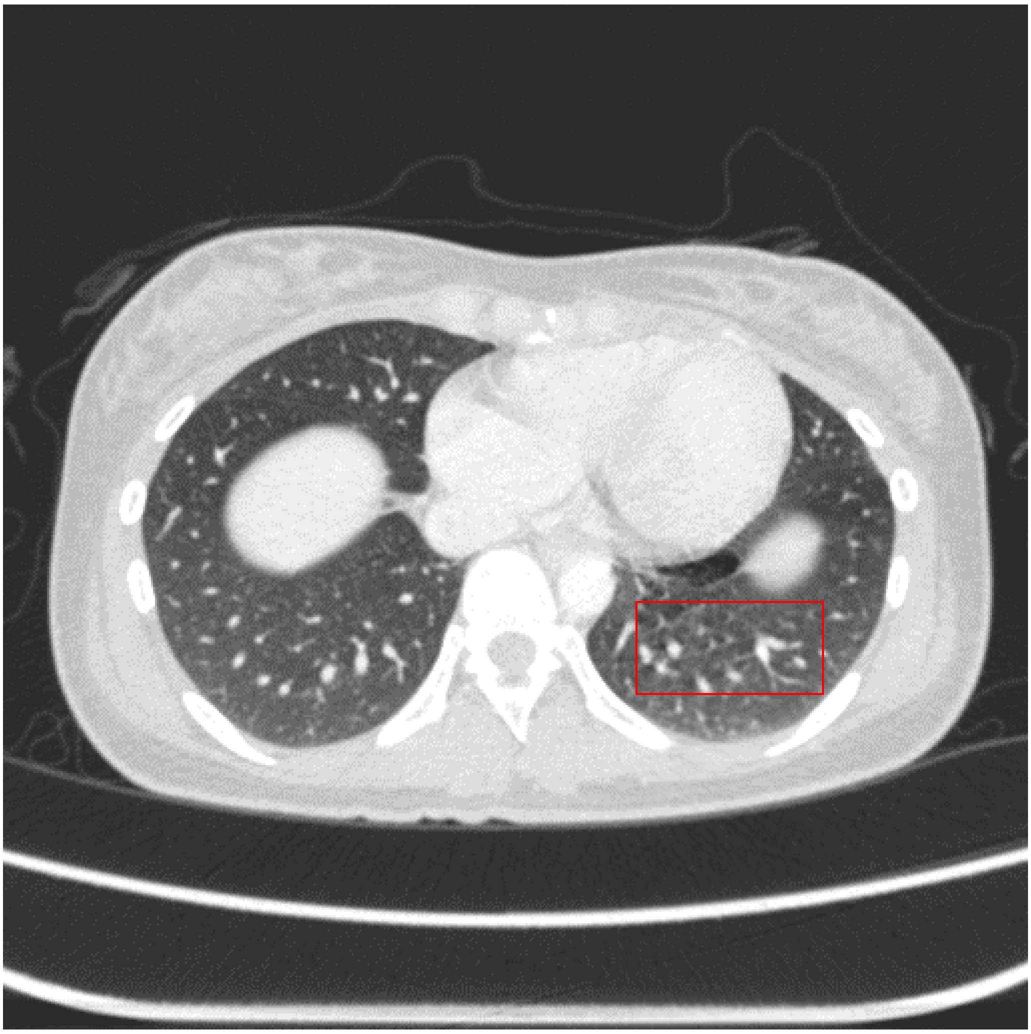

Supplementary Figure S24. FBP image of motion artifacts (Case F-M2). L/W: -500/1500 HU.

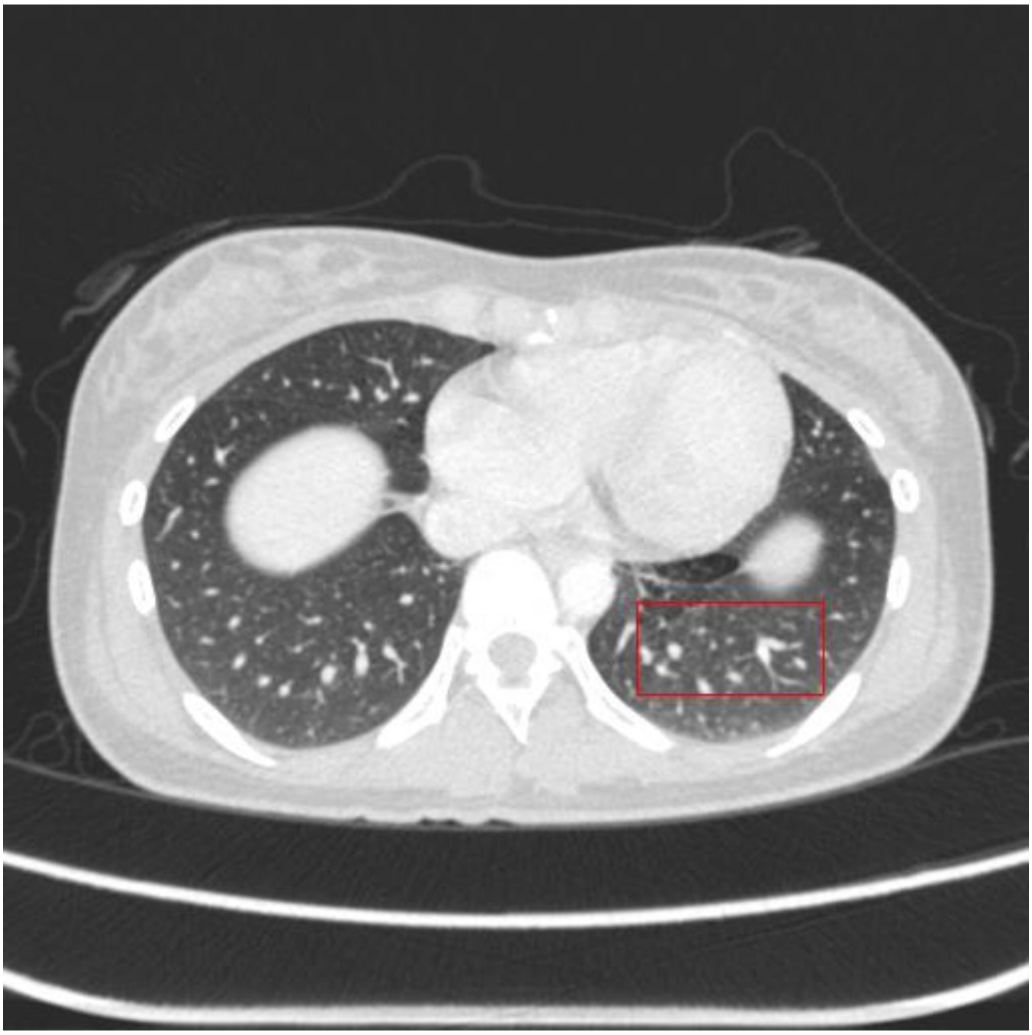

Supplementary Figure S25. SCULLI-TX image of Case F-M2. L/W: -500/1500 HU.

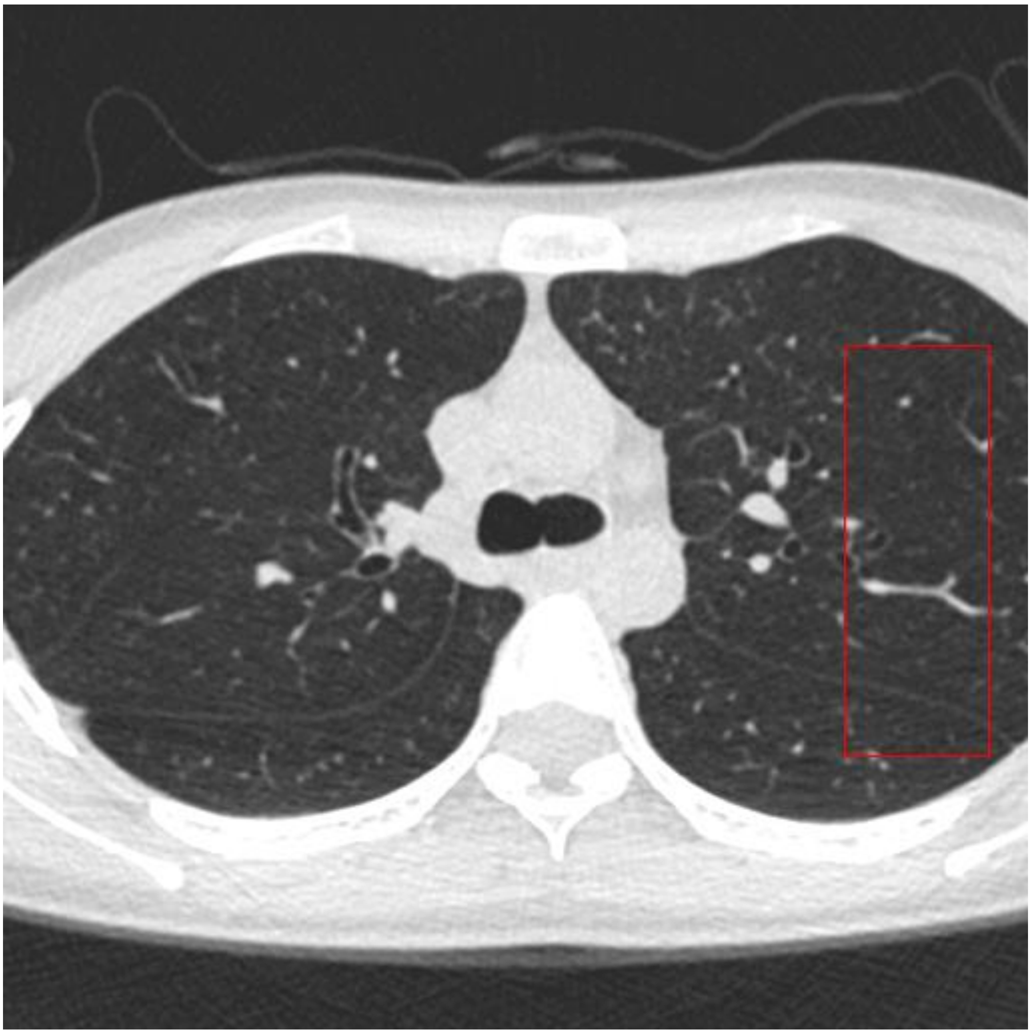

Supplementary Figure S26. FBP image of no motion artifacts (Case A-S1). L/W: -500/1500 HU.

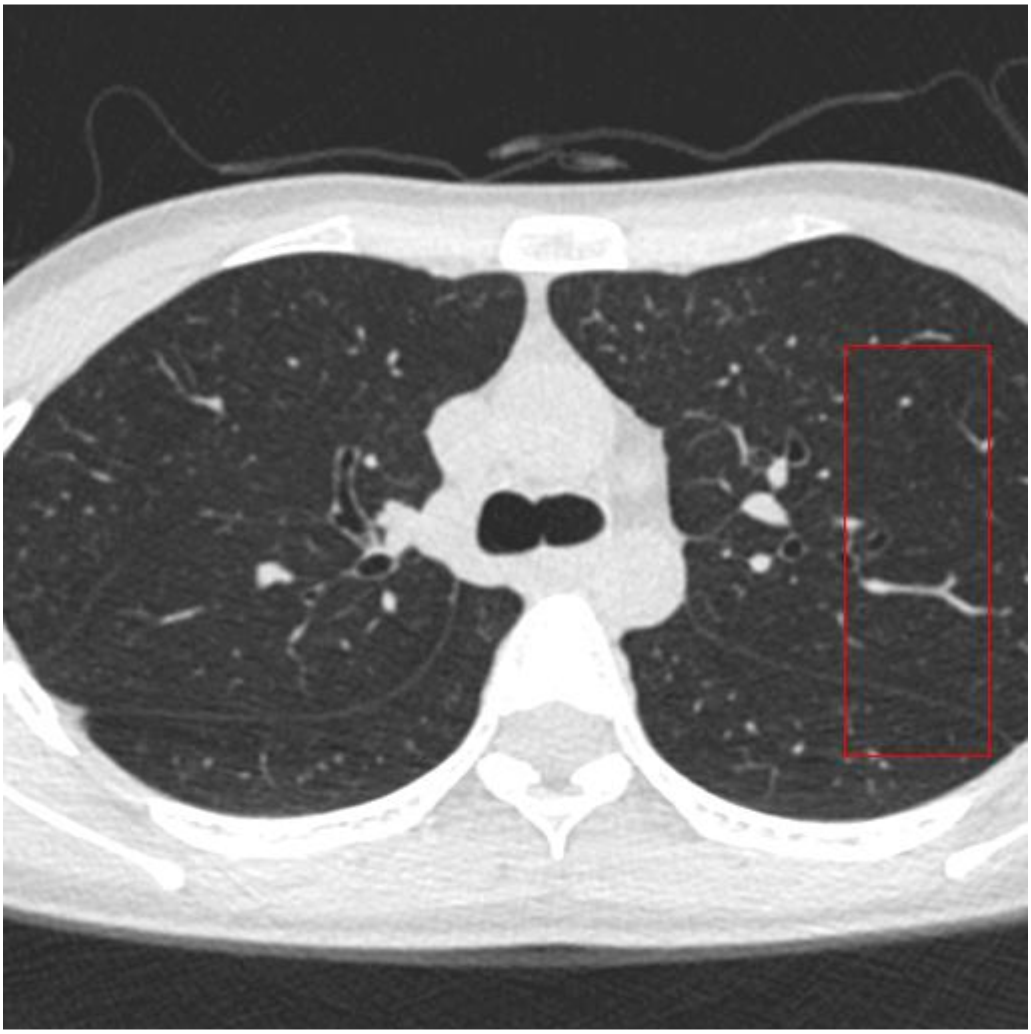

Supplementary Figure S27. SCULLI-TX image of Case A-S1. L/W: -500/1500 HU.

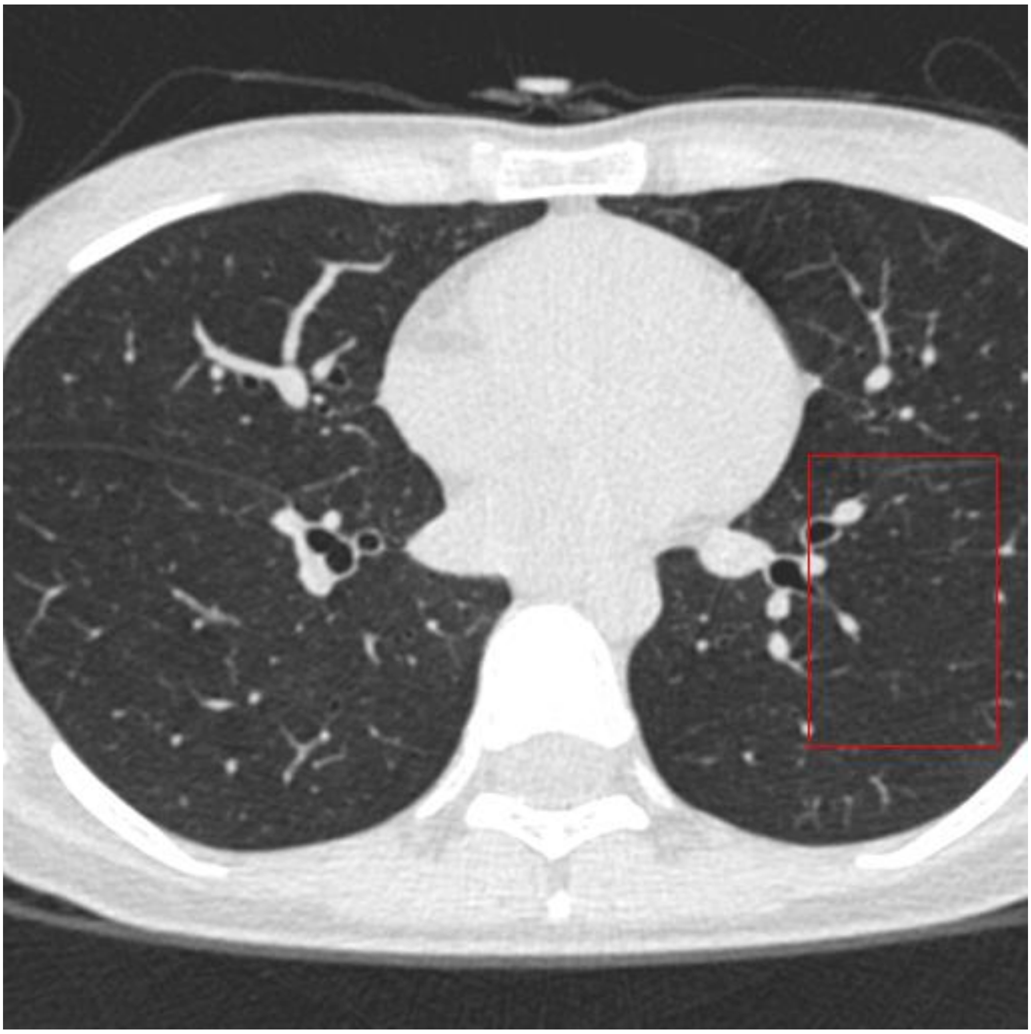

Supplementary Figure S28. FBP image of no motion artifacts (Case A-S2). L/W: -500/1500 HU.

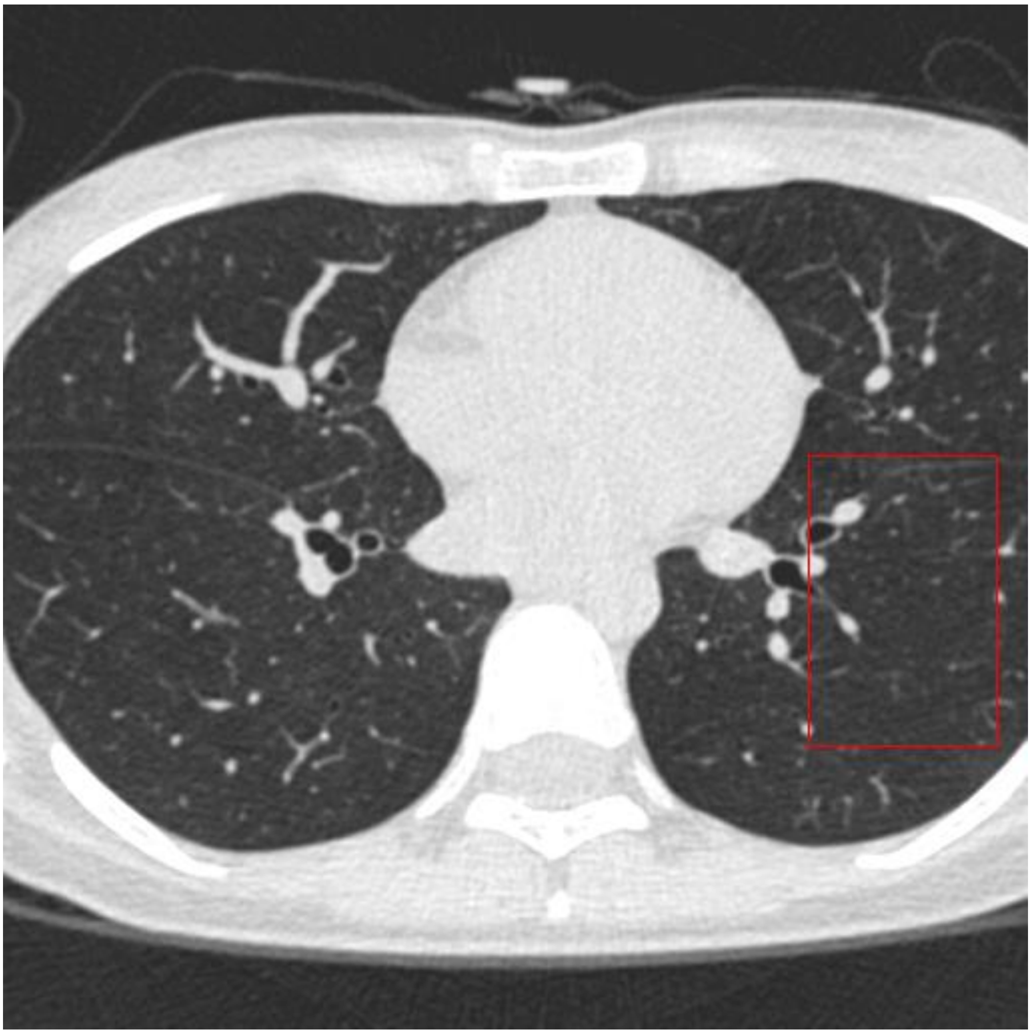

Supplementary Figure S29. SCULLI-TX image of Case A-S2. L/W: -500/1500 HU.

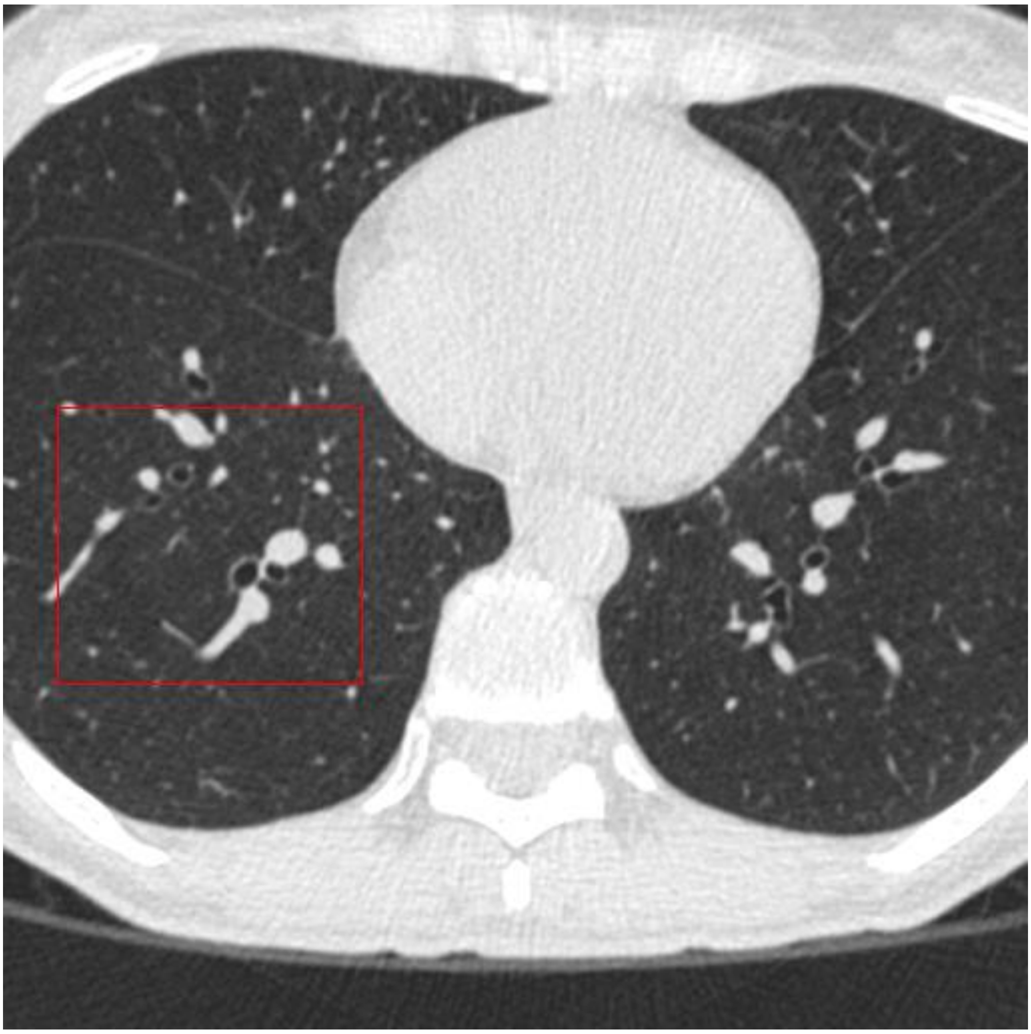

Supplementary Figure S30. FBP image of no motion artifacts (Case B-S1). L/W: -500/1500 HU.

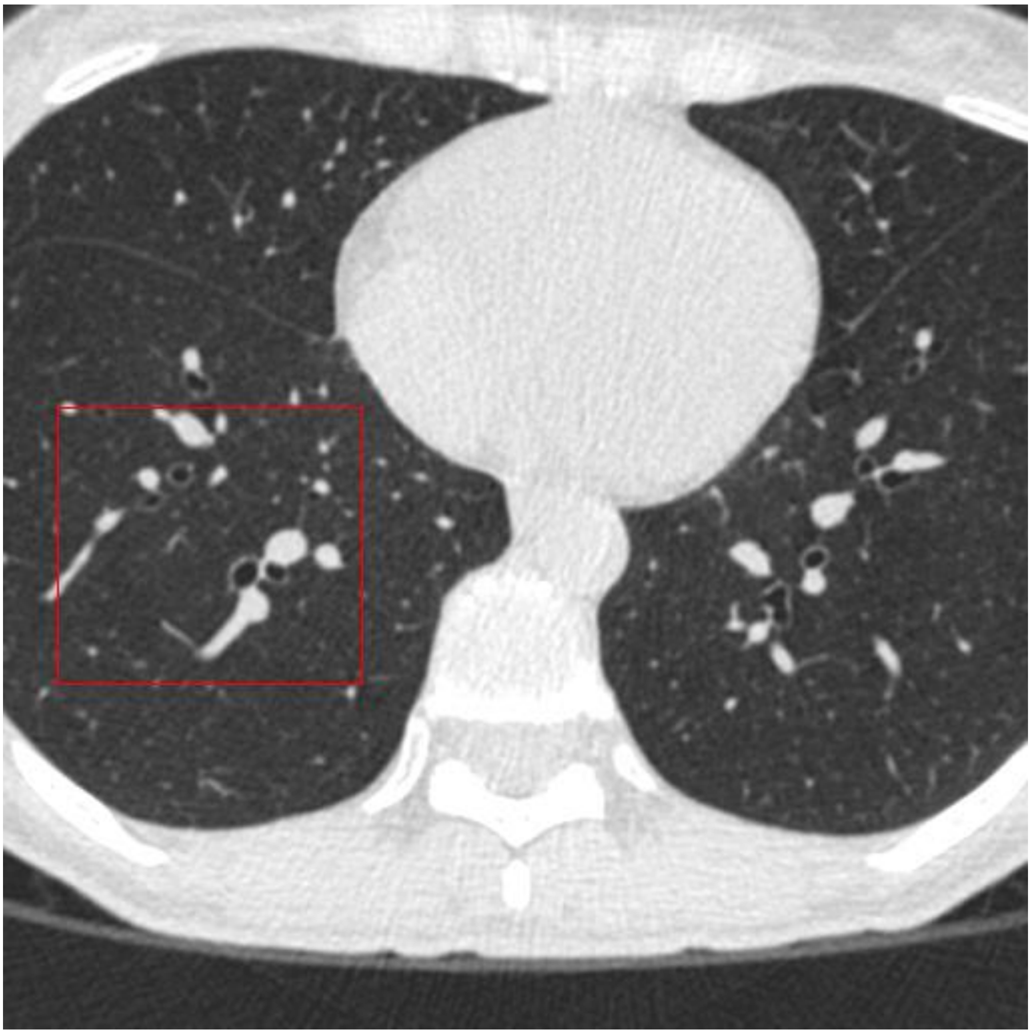

Supplementary Figure S31. SCULLI-TX image of Case B-S1. L/W: -500/1500 HU.

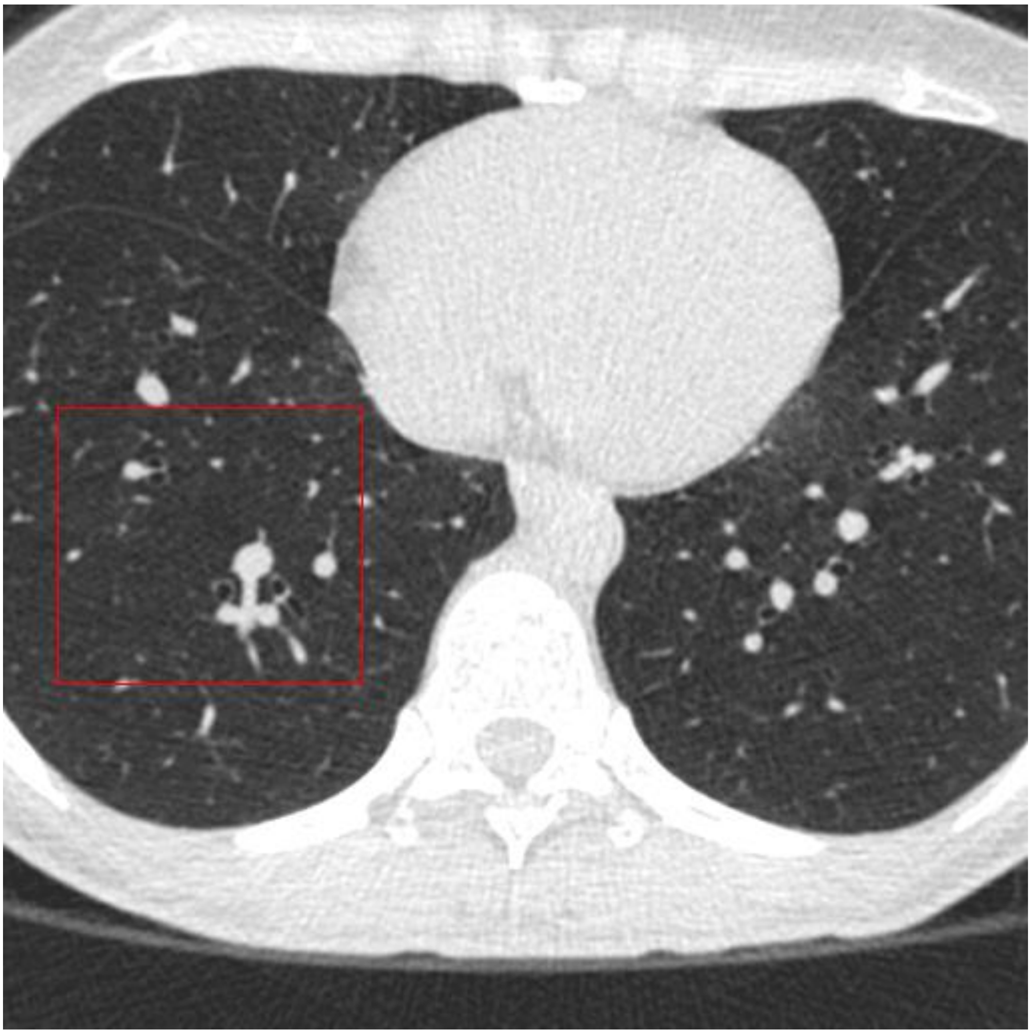

Supplementary Figure S32. FBP image of no motion artifacts (Case B-S2). L/W: -500/1500 HU.

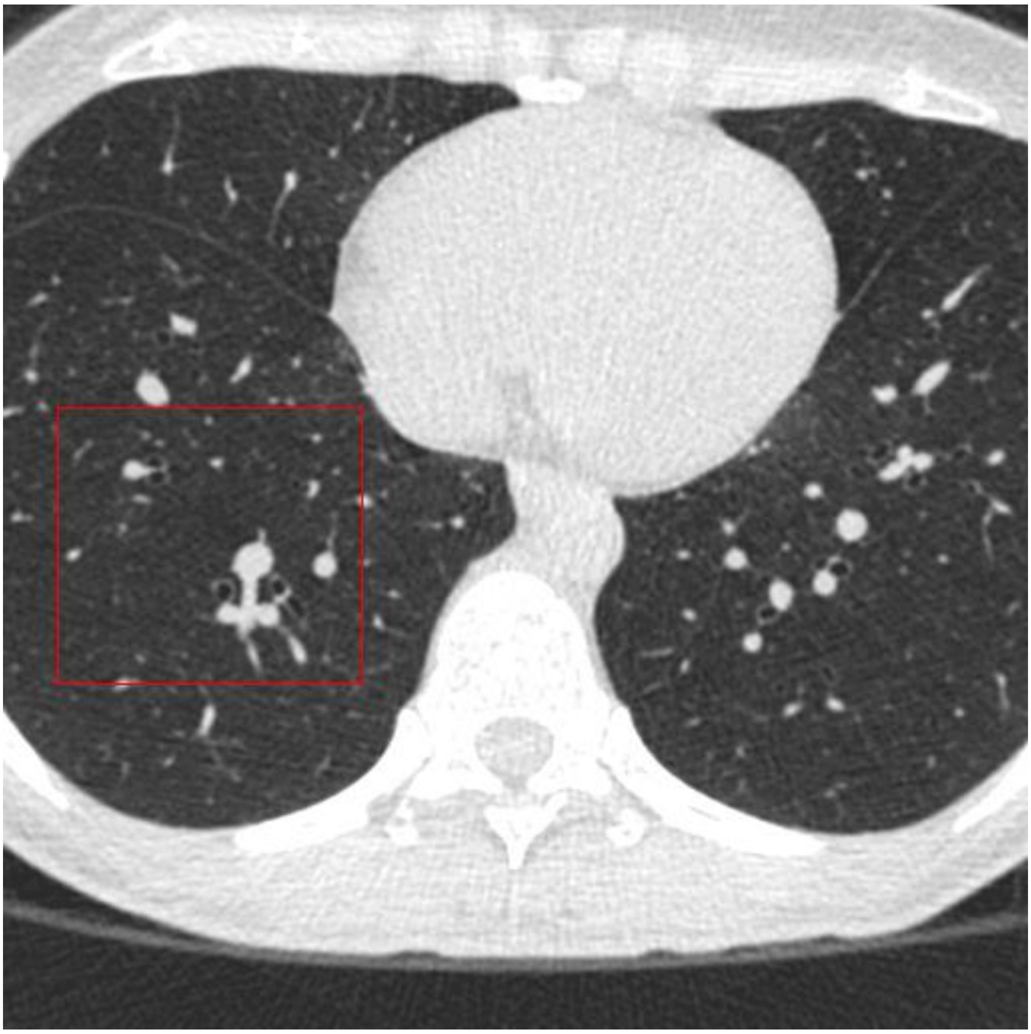

Supplementary Figure S33. SCULLI-TX image of Case B-S2. L/W: -500/1500 HU.

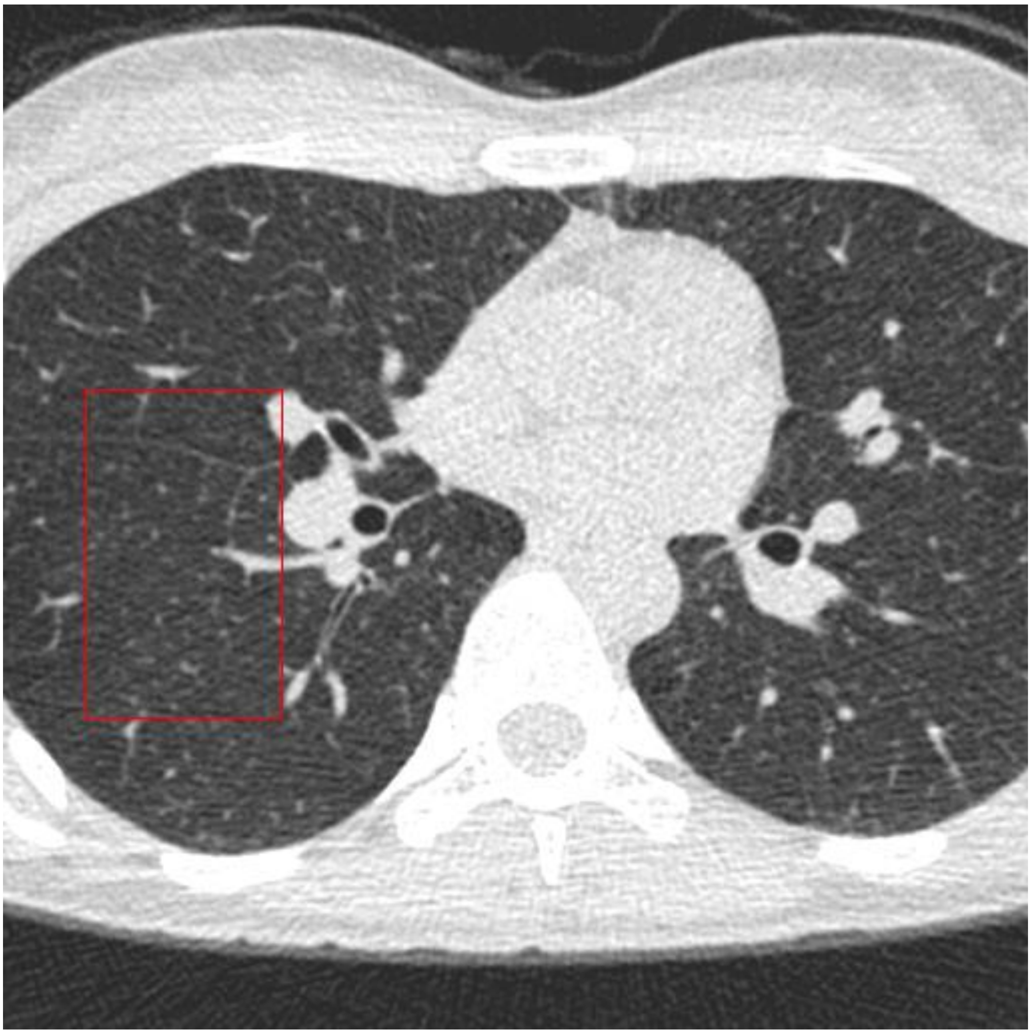

Supplementary Figure S34. FBP image of no motion artifacts (Case C-S1). L/W: -500/1500 HU.

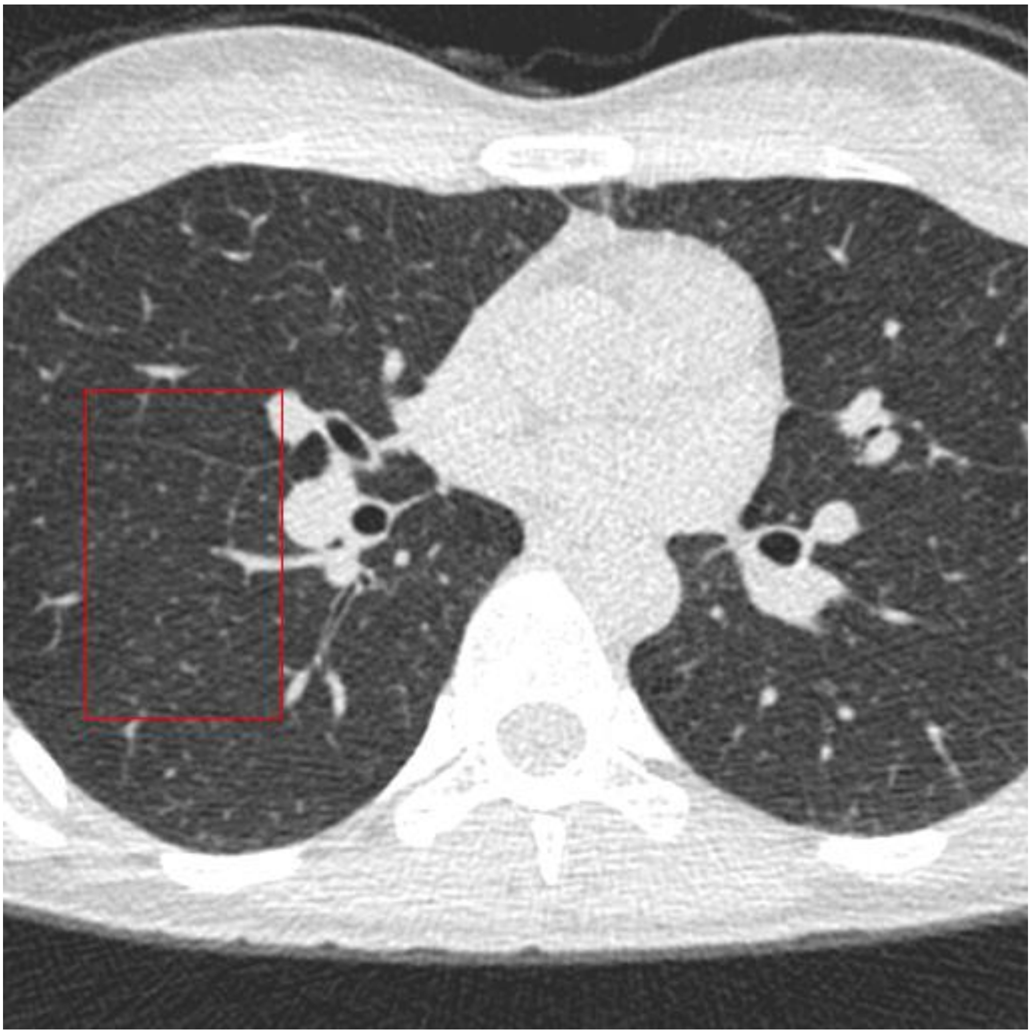

Supplementary Figure S35. SCULLI-TX image of Case C-S1. L/W: -500/1500 HU.

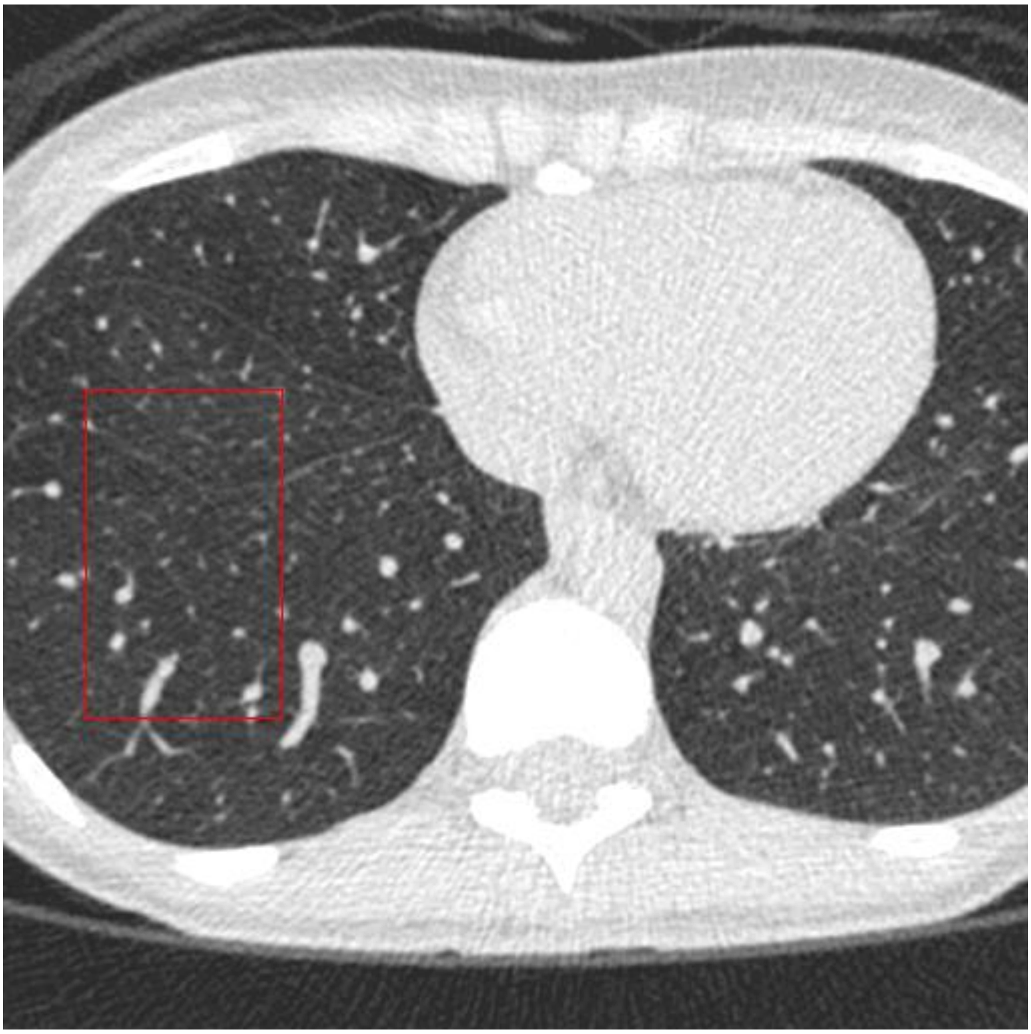

Supplementary Figure S36. FBP image of no motion artifacts (Case C-S2). L/W: -500/1500 HU.

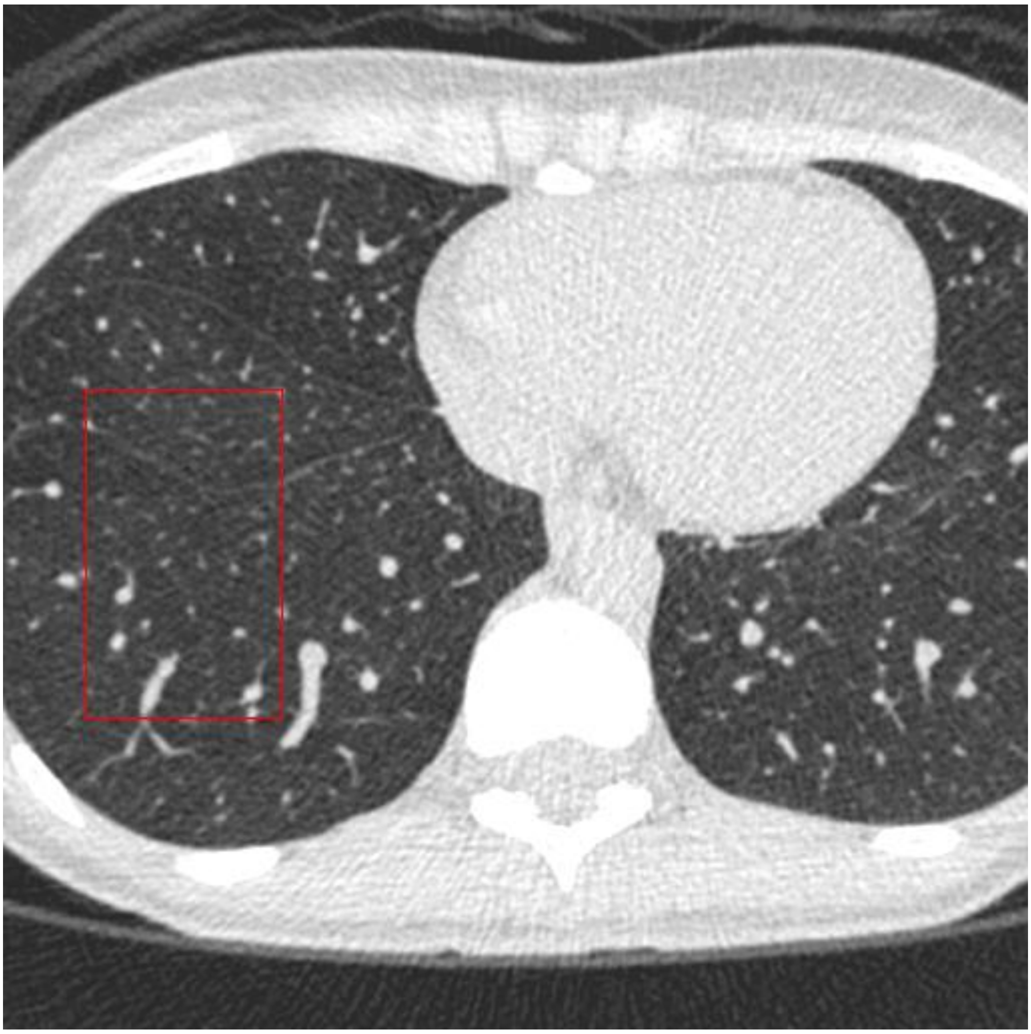

Supplementary Figure S37. SCULLI-TX image of Case C-S2. L/W: -500/1500 HU.

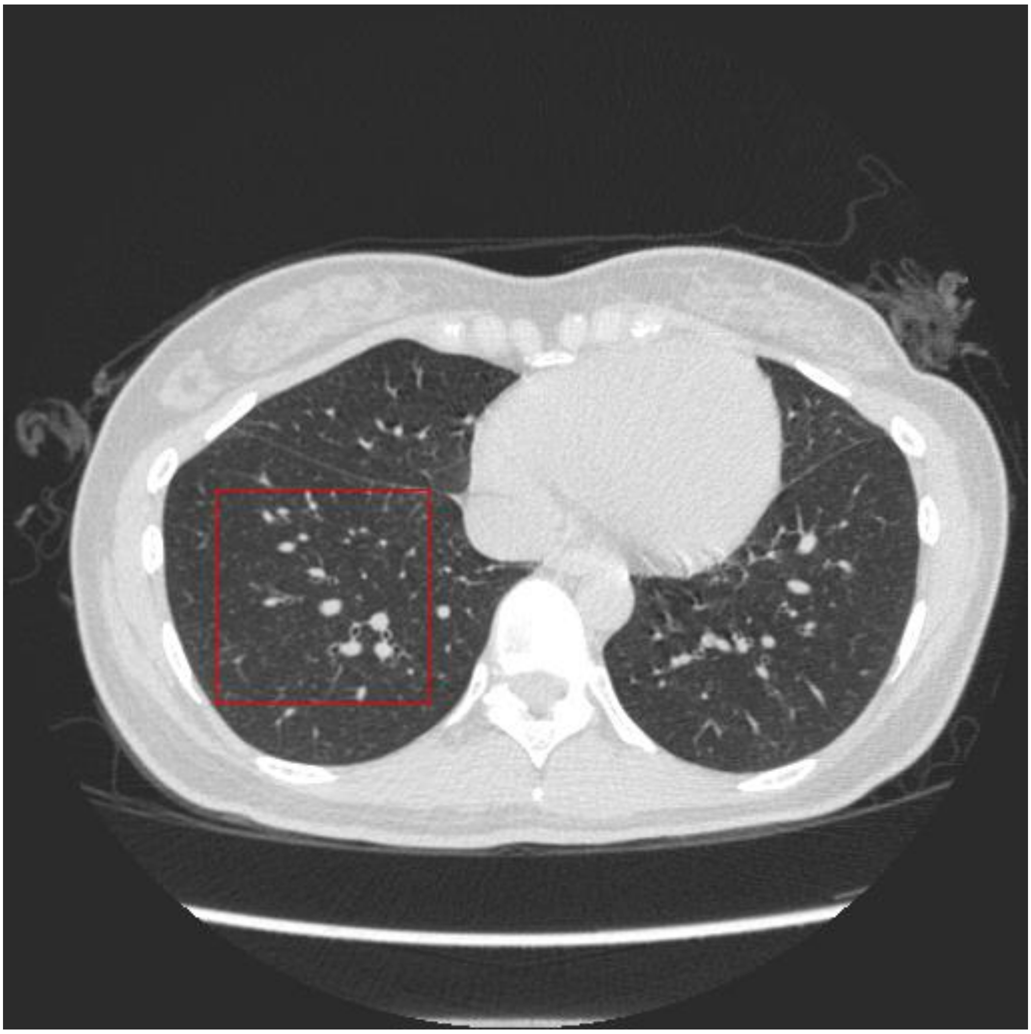

Supplementary Figure S38. FBP image of no motion artifacts (Case D-S1). L/W: -500/1500 HU.

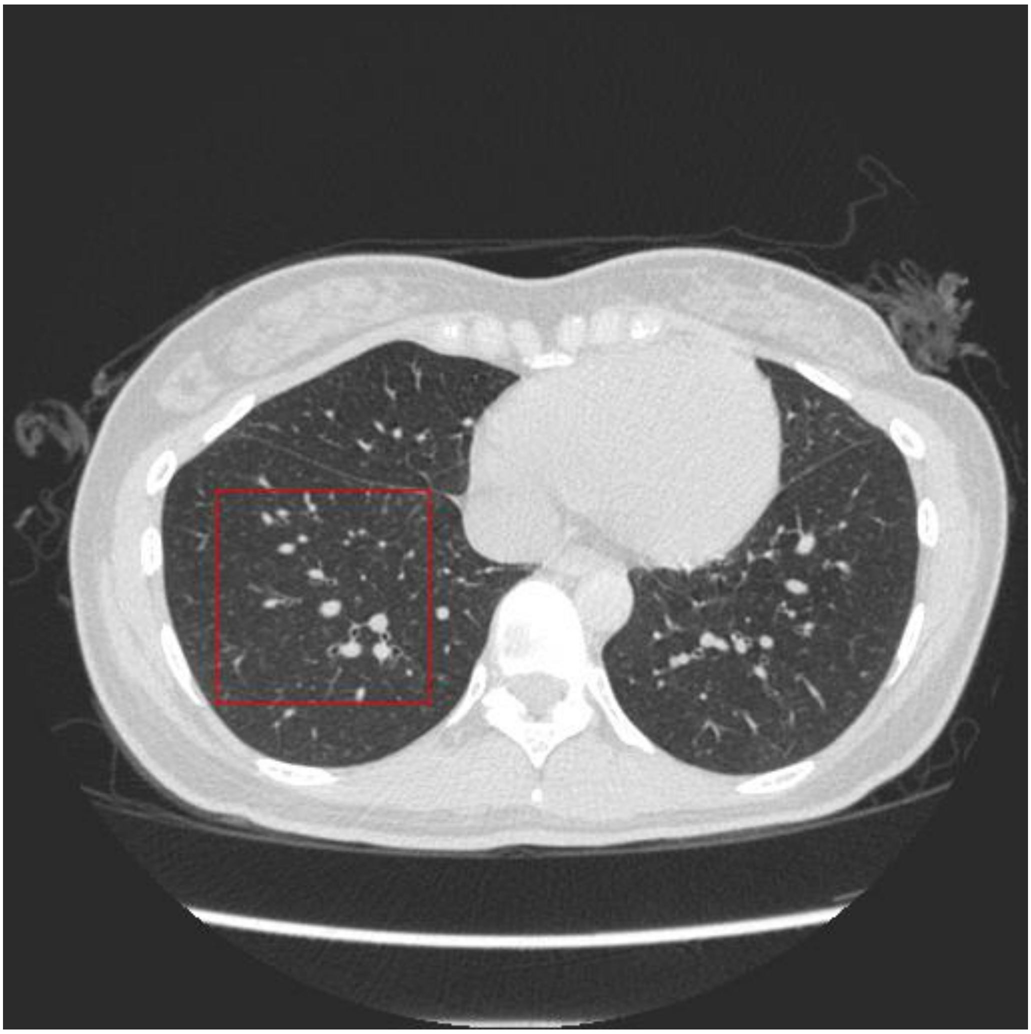

Supplementary Figure S39. SCULLI-TX image of Case D-S1. L/W: -500/1500 HU.

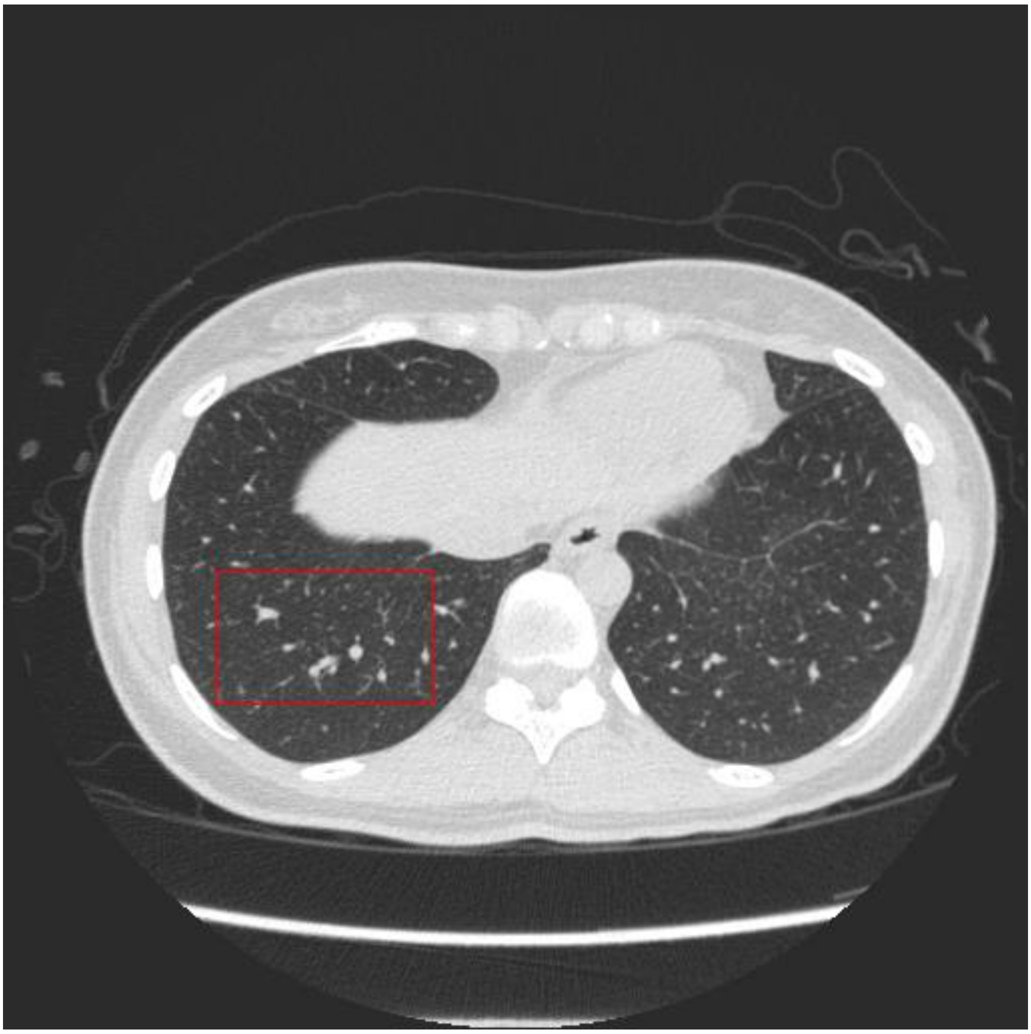

Supplementary Figure S40. FBP image of no motion artifacts (Case D-S2). L/W: -500/1500 HU.

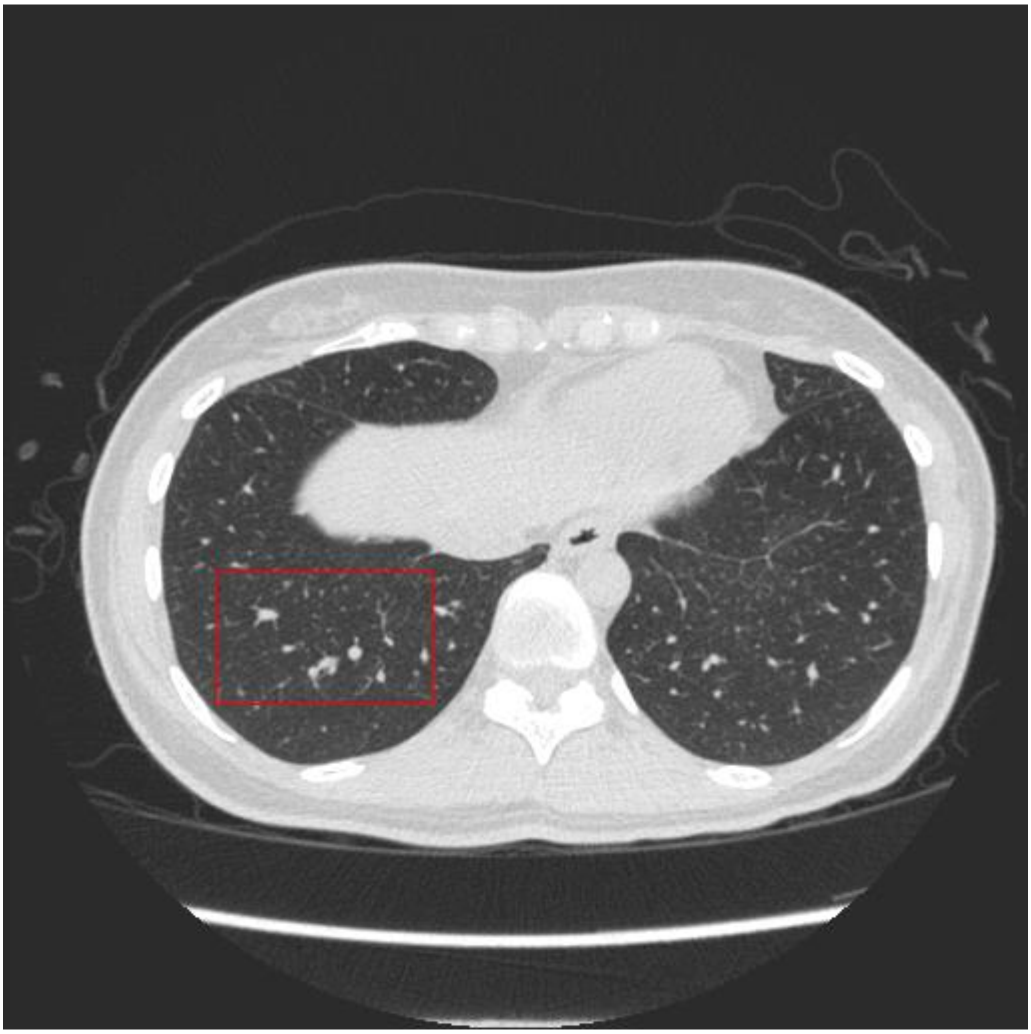

Supplementary Figure S41. SCULLI-TX image of Case D-S2. L/W: -500/1500 HU.

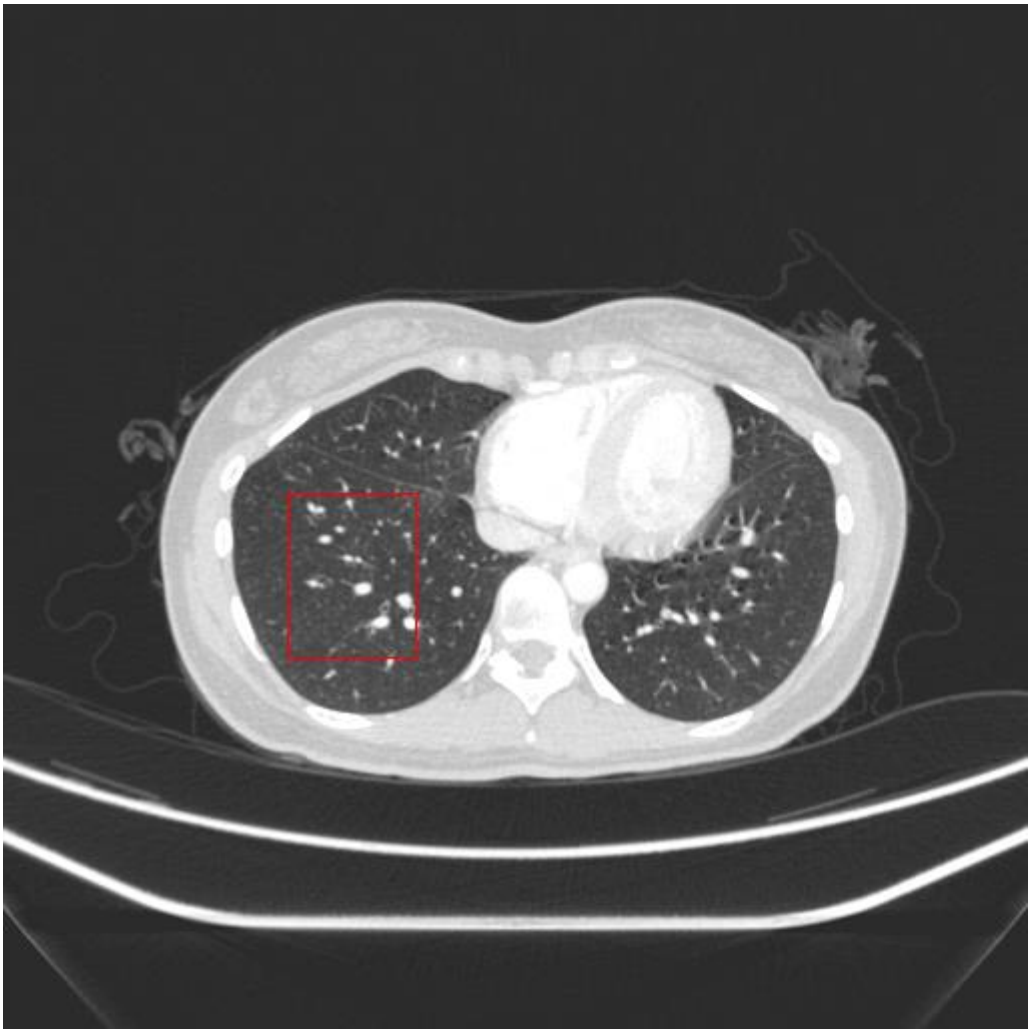

Supplementary Figure S42. FBP image of no motion artifacts (Case E-S1). L/W: -500/1500 HU.

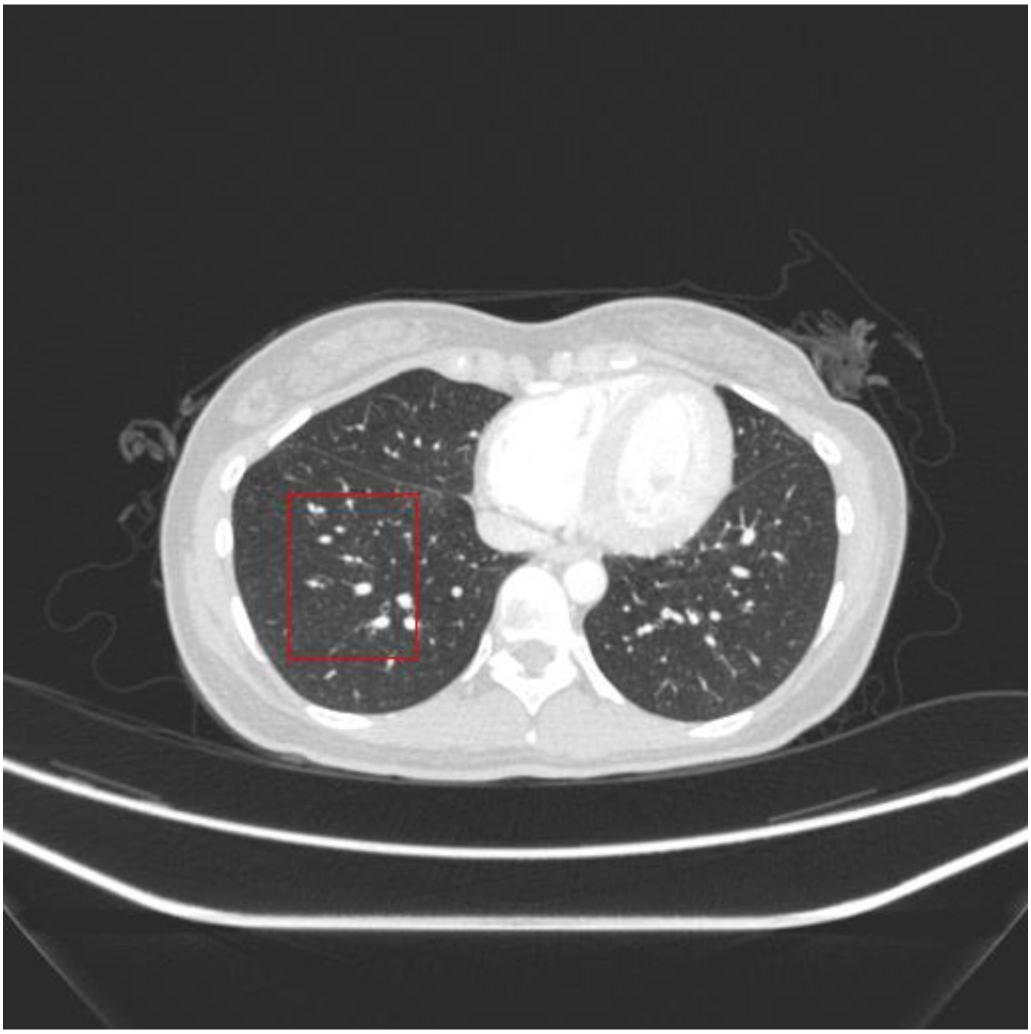

Supplementary Figure S43. SCULLI-TX image of Case E-S1. L/W: -500/1500 HU.

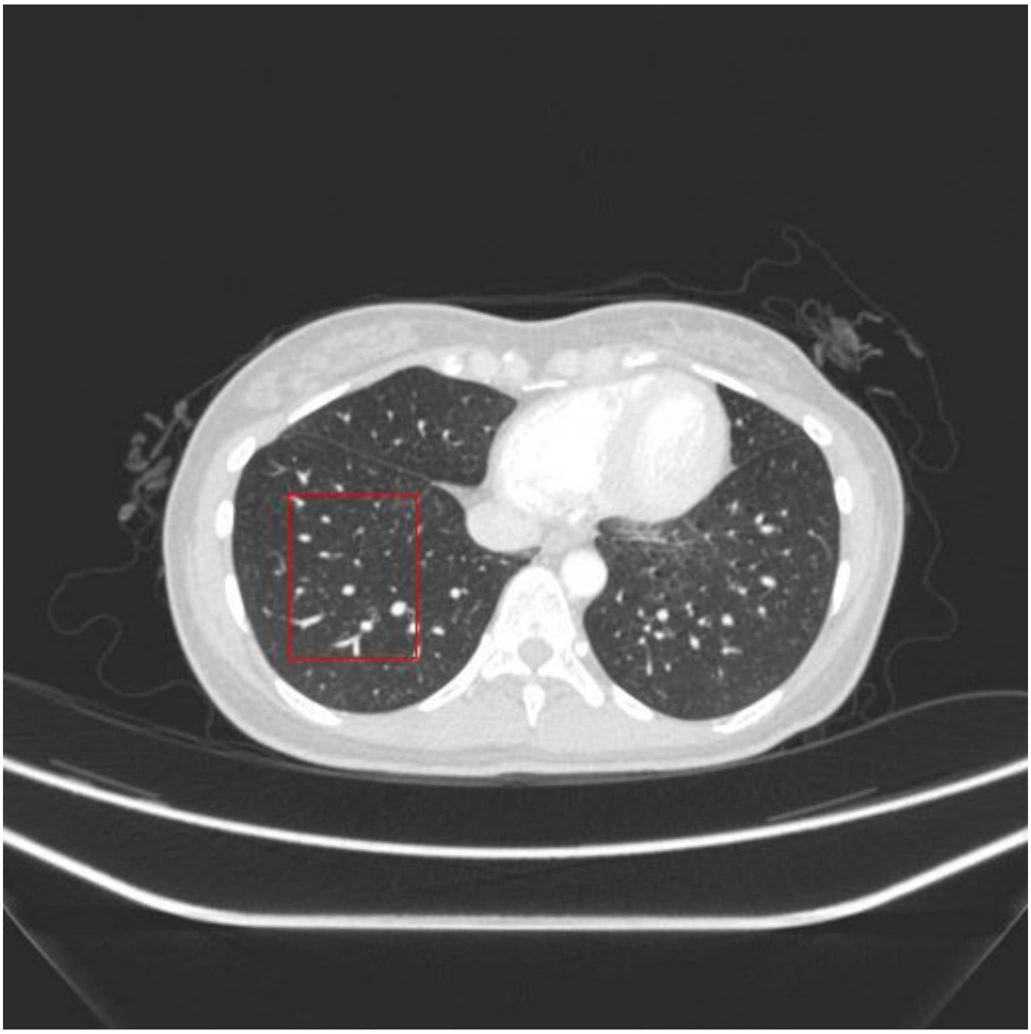

Supplementary Figure S44. FBP image of no motion artifacts (Case E-S2). L/W: -500/1500 HU.

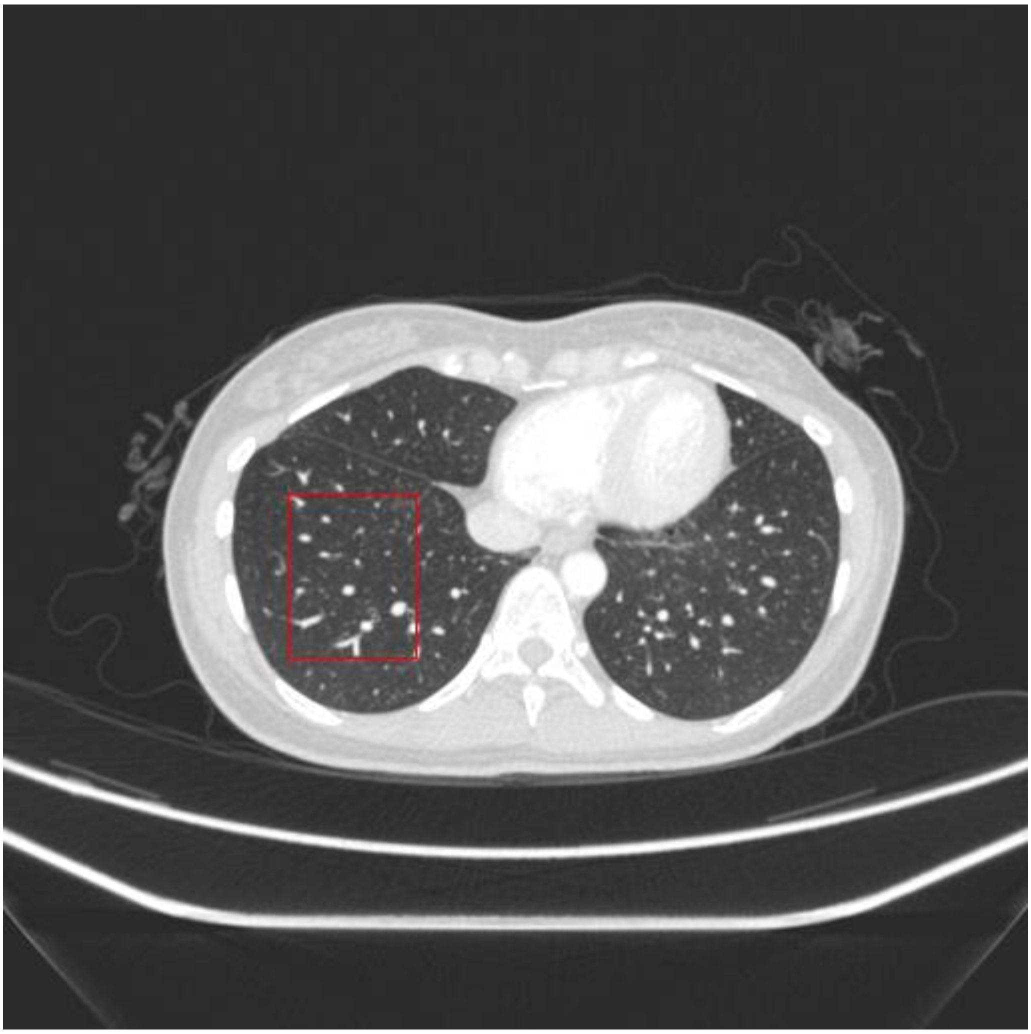

Supplementary Figure S45. SCULLI-TX image of Case E-S2. L/W: -500/1500 HU.

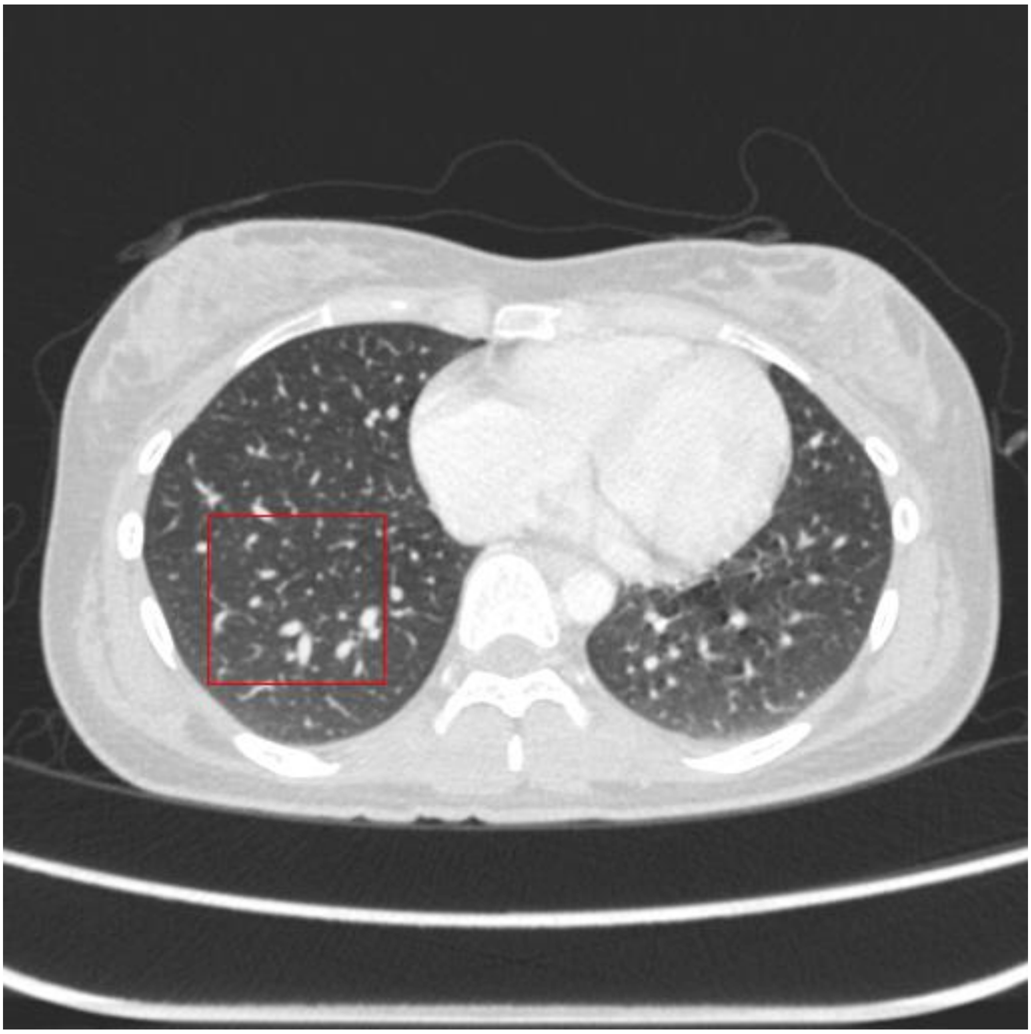

Supplementary Figure S46. FBP image of no motion artifacts (Case F-S1). L/W: -500/1500 HU.

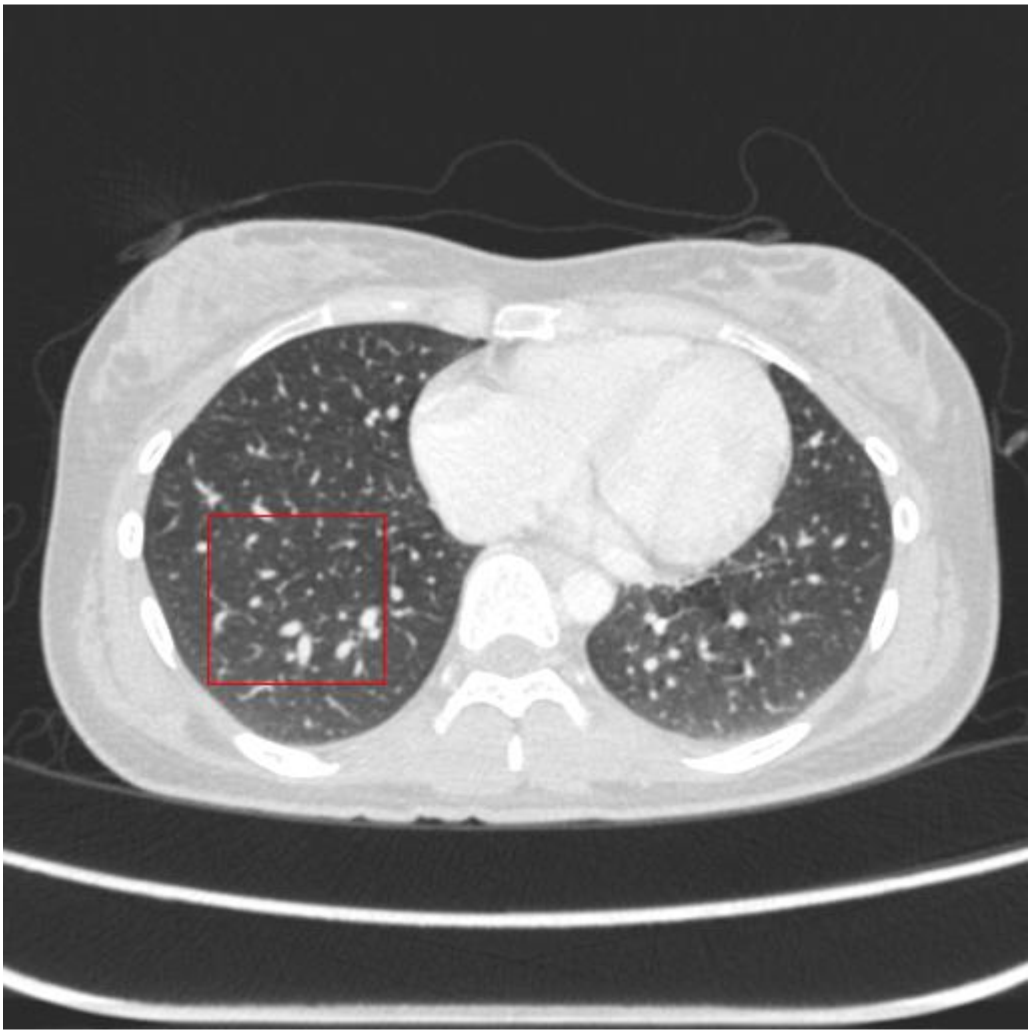

Supplementary Figure S47. SCULLI-TX image of Case F-S1. L/W:-500/1500 HU.

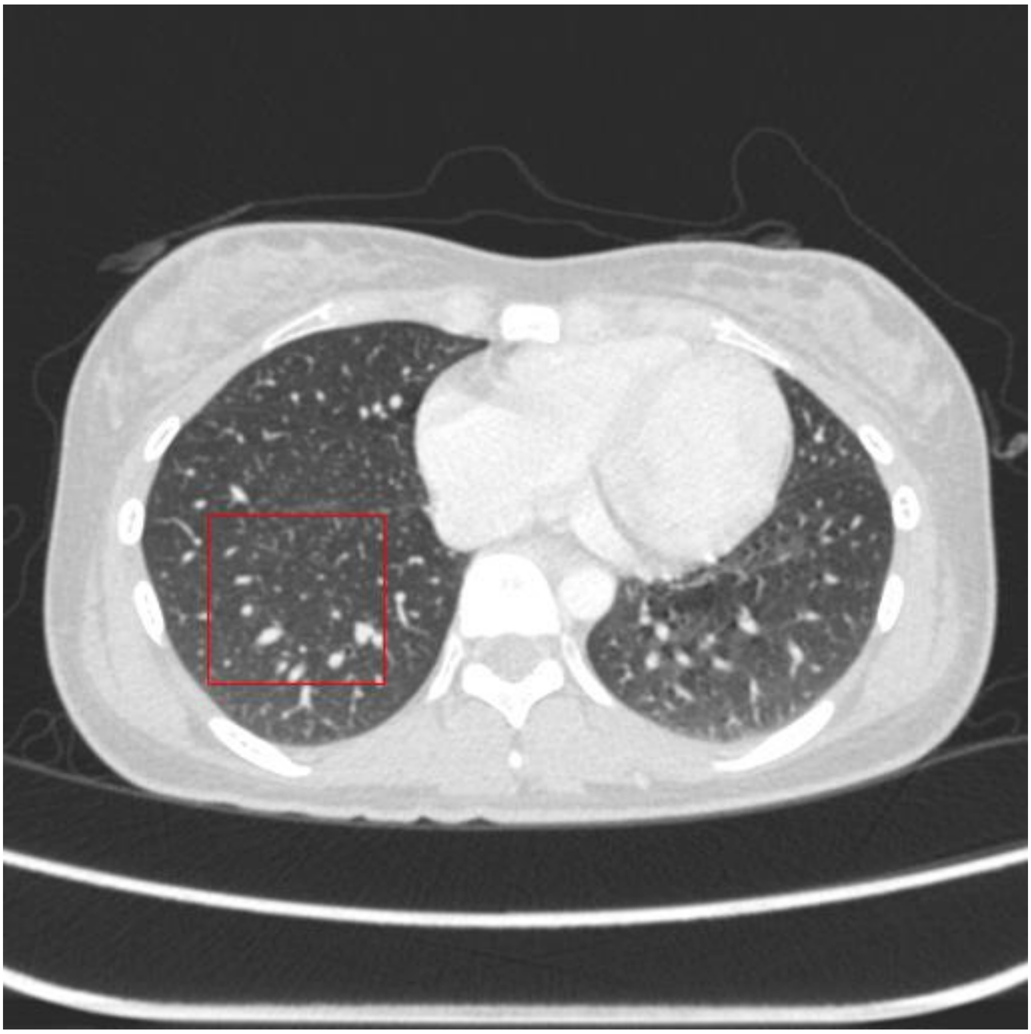

Supplementary Figure S48. FBP image of no motion artifacts (Case F-S2). L/W: -500/1500 HU.

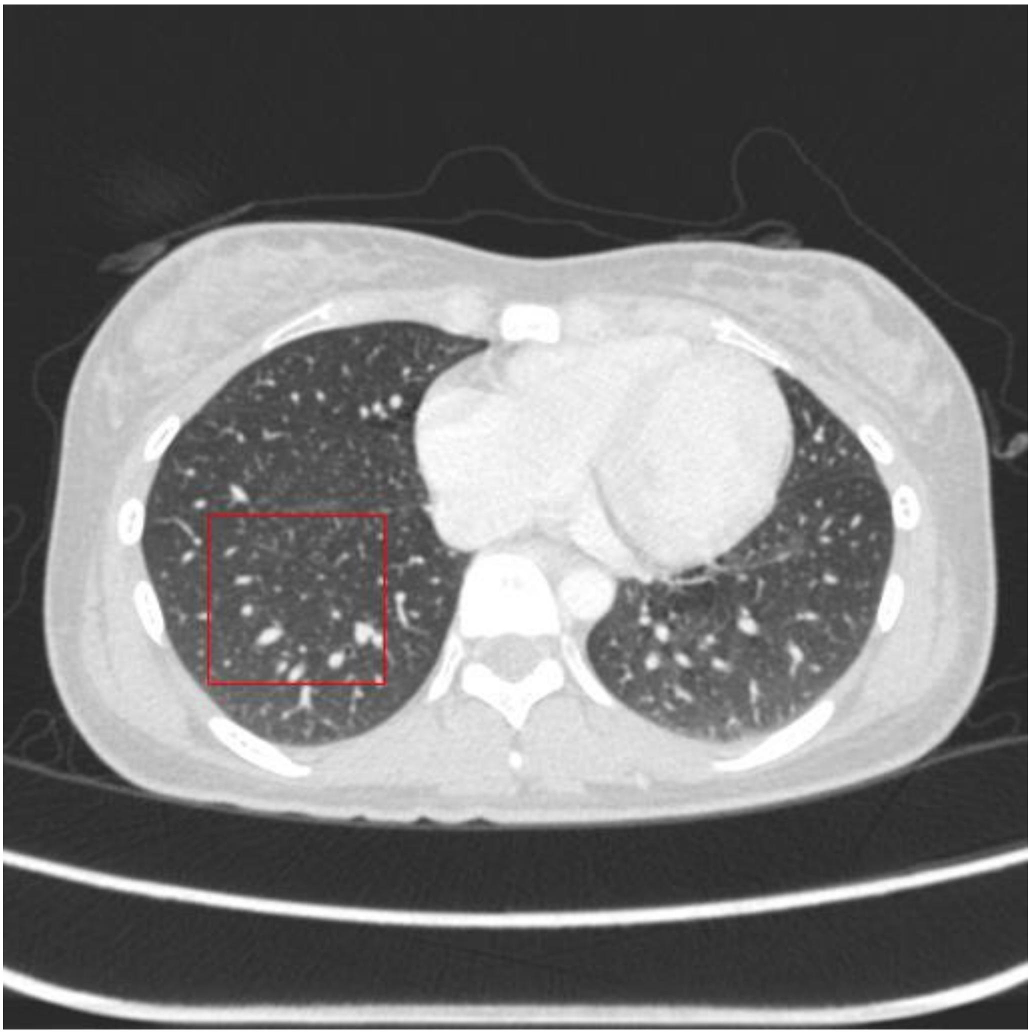

Supplementary Figure S49. SCULLI-TX image of Case F-S2. L/W: -500/1500 HU.
